# The Potential Roles of Mucosa-Associated Invariant T Cells in the Pathogenesis of Gut Graft-Versus-Host Disease After Hematopoietic Stem Cell Transplantation

**DOI:** 10.3389/fimmu.2021.720354

**Published:** 2021-09-03

**Authors:** Meng-Ge Gao, Yan Hong, Xiang-Yu Zhao, Xin-An Pan, Yu-Qian Sun, Jun Kong, Zhi-Dong Wang, Feng-Rong Wang, Jing-Zhi Wang, Chen-Hua Yan, Yu Wang, Xiao-Jun Huang, Xiao-Su Zhao

**Affiliations:** ^1^Peking University People’s Hospital, Peking University Institute of Hematology, National Clinical Research Center for Hematologic Disease, Beijing Key Laboratory of Hematopoietic Stem Cell Transplantation, Beijing, China; ^2^Collaborative Innovation Center of Hematology, Peking University, Beijing, China; ^3^Research Unit of Key Technique for Diagnosis and Treatments of Hematologic Malignancies, Chinese Academy of Medical Sciences, Beijing, China; ^4^Peking-Tsinghua Center for Life Sciences, Beijing, China

**Keywords:** mucosa-associated invariant T cell, allo-HSCT, gut acute graft-versus-host disease, intestinal flora, immunomodulatory

## Abstract

Gut acute graft-versus-host disease (aGVHD) is a serious complication after allogeneic hematopoietic stem cell transplantation (allo-HSCT) and is associated with high mortality. Mucosa-associated invariant T (MAIT) cells are a group of innate-like T cells enriched in the intestine that can be activated by riboflavin metabolites from various microorganisms. However, little is known about the function or mechanism of action of MAIT cells in the occurrence of gut aGVHD in humans. In our study, multiparameter flow cytometry (FCM) was used to evaluate the number of MAIT cells and functional cytokines. 16S V34 region amplicon sequencing analysis was used to analyze the intestinal flora of transplant patients. *In vitro* stimulation and coculture assays were used to study the activation and function of MAIT cells. The number and distribution of MAIT cells in intestinal tissues were analyzed by immunofluorescence technology. Our study showed that the number and frequency of MAIT cells in infused grafts in gut aGVHD patients were lower than those in no-gut aGVHD patients. Recipients with a high number of MAITs in infused grafts had a higher abundance of intestinal flora in the early posttransplantation period (+14 days). At the onset of gut aGVHD, the number of MAIT cells decreased in peripheral blood, and the activation marker CD69, chemokine receptors CXCR3 and CXCR4, and transcription factors Rorγt and T-bet tended to increase. Furthermore, when gut aGVHD occurred, the proportion of MAIT17 was higher than that of MAIT1. The abundance of intestinal flora with non-riboflavin metabolic pathways tended to increase in gut aGVHD patients. MAIT cells secreted more granzyme B, tumor necrosis factor (TNF)-α, and interferon (IFN)-γ under the interleukin (IL)-12/IL-18 stimulation [non-T-cell receptor (TCR) signal] and secreted most of the IL-17 under the cluster of differentiation (CD)3/CD28 stimulation (TCR signal). MAIT cells inhibited the proliferation of CD4+ T cells *in vitro*. In conclusion, the lower number of MAIT cells in infused grafts was related to the higher incidence of gut aGVHD, and the number of MAIT cells in grafts may affect the composition of the intestinal flora of recipients early after transplantation. The flora of the riboflavin metabolism pathway activated MAIT cells and promoted the expression of intestinal protective factors to affect the occurrence of gut aGVHD in humans.

## Introduction

Gut acute graft-versus-host disease (aGVHD) is a serious complication after allogeneic hematopoietic stem cell transplantation (allo-HSCT) and is associated with high mortality (1–5). Its pathogenesis mainly involves the activation of donor T cells, which cause cytokine storm-inflammatory damage and attack the host’s intestinal tissues (2, 3). Growing evidence shows that gut microbiota can affect the occurrence and development of gut aGVHD (6–11). Systemic irradiation and chemotherapy before allo-HSCT can cause intestinal mucosal damage, resulting in the loss of integrity of the intestinal epithelial barrier. These injuries can also lead to the release of interleukin (IL)-17 and other protective cytokines induced by microbiogenic antigens whose main function is to limit the transmission of pathogens (1, 12–14). For patients with gut aGVHD, as further damage to the integrity of the intestinal epithelium is caused by aGVHD, the intestinal flora becomes ecologically unbalanced, and the diversity of the flora is significantly reduced (6–8). Previous studies have shown that in allo-HSCT recipients, aGVHD-induced intestinal inflammation is associated with changes in the metabolites produced by the intestinal flora, which in turn influence the severity of intestinal inflammation by regulating immune cells (6–10).

Mucosa-associated invariant T (MAIT) cells are a group of innate-like T cells that express the semi-invariant T-cell receptor (TCR) Vα7.2-Jα33 and the C-type lectin-like receptor CD161 in humans and are restricted by non-polymorphic major histocompatibility complex (MHC)-related molecule 1 (MR1). The distribution of MAIT cells is tissue-specific and enriched in mucosal tissues such as the liver (20%–50% of liver T cells), intestine (10% of intestinal lamina propria T cells), and peripheral blood (PB) (1%–10% of T cells) (12, 15–17). The riboflavin derivatives (vitamin B2) of some flora can bind to MR1 molecules to activate MAIT cells by the MR1-dependent pathway (TCR-dependent pathway) (18–21). In addition, the high expression of multiple cytokine receptors on the surface of MAIT cells (especially IL-12R and IL-18R) causes MAIT cells to be activated by cytokine stimulation (non-TCR-dependent pathway) (22–26). According to the expressed transcription factors T-bet and RORγt, MAIT cells can be divided into two mature functional subsets, MAIT1 and MAIT17 (15, 17, 18, 27, 28). Different MAIT subsets can migrate to the site of action by expressing different chemokine receptors, such as C-C chemokine receptor (CCR)6 and C-X-C chemokine receptor (CXCR)3, and then play different functional roles by expressing cytokines, such as tumor necrosis factor (TNF)-α, interferon (IFN)-γ, IL-17, and granzyme B (GrB) (22–27).

Previous studies have found that the rapid reconstitution of MAIT cells after transplantation is associated with the number of MAIT cells in grafts and the abundance of certain intestinal flora (such as *Blautia* and *Bifidobacterium*) (8, 29–32). However, there is currently no direct evidence that the number of MAIT cells is related to the abundance of flora with riboflavin metabolic pathways. Furthermore, our previous studies confirmed that the CD8+CD161^hi^ cell population (MAIT cells account for more than 90% of CD8+CD161^hi^ cells) is related to the occurrence of aGVHD, especially gut aGVHD (33, 34). Taken together, previous reports have found a correlation between MAIT cells or intestinal flora and the occurrence of aGVHD. However, no report has shown how MAIT cells play a role in the pathogenesis of human gut aGVHD. They may protect the intestinal mucosa from further damage attributed to inflammation or the immune response by altering the composition of the intestinal flora or exerting certain immunoregulatory effects, which needs further research to confirm.

In our study, we collected continuous samples from 150 transplanted patients and comprehensively investigated the relationship among the changes in MAIT cell function, intestinal flora composition, and the occurrence of gut aGVHD in humans. This study found for the first time that the number of MAIT cells in infused grafts could affect the occurrence of gut aGVHD and that the intestinal flora of the riboflavin metabolism pathway may participate in the regulation of MAIT cells on gut aGVHD through TCR-dependent signals and preliminarily explored the potential mechanism by which MAIT cells affect the occurrence of gut aGVHD in humans.

## Materials and Methods

### Patients and Sample Collection

The study was approved by the ethics committee of Peking University People’s Hospital (Ethics number 2018PHB222-01) and was carried out after obtaining the consent of the patients or legal guardian (Details in the **Supplementary Material and Methods**).

For multiparameter flow cytometry (FCM) analysis and 16S rRNA V3-V4 region for high-throughput sequencing, the enrolled 150 consecutive patients who underwent allo-HSCT in our institute from March 1, 2019, to December 1, 2019, and met the above criteria included 116 unmanipulated haploidentical HSCT (haplo-HSCT) and 34 human leukocyte antigen (HLA)-matched sibling donor transplantation (MSDT) (**Supplementary Table S1**).

### Flow Cytometry

FCM was used to detect the frequency and number of MAIT cells in grafts and PB and the expression of functional factors of MAIT cells. In the study, MAIT cells were defined in the group of cells that gated on CD161+Vα7.2+ in CD3+T cells (CD3+CD161+ Vα7.2+), and its frequency was defined as the ratio of CD161+Vα7.2+ to CD3+T cells (CD161+Vα7.2+/CD3+). Conventional T (Tc) and regulatory T (Treg) cells were defined as a group of cells that expressed CD3+CD4+CD25- and CD3+CD4+CD25+CD127-, respectively (Details in the **Supplementary Material and Methods**).

### Mucosa-Associated Invariant T Cell Isolation

The PB came from three healthy adults, and peripheral blood mononuclear cells (PBMCs) were separated with lymphocyte separation fluid (Ficol). MAIT cells (CD3+CD161+Vα7.2+) were isolated from PBMCs using fluorescence-activated cell sorting (FACS)—sorted (FACS Aria II, BD Biosciences, USA) with antibodies CD3 (Percp, Biolegend, USA), CD161 (PE-CY7, Biolegend, USA), Vα7.2 (PE, Biolegend, USA) according to the manufacturer’s instructions, which included one step of positive selection for CD3+ cells and next step of double positive selection for CD161+ and Vα7.2+ cells.

### *In Vitro* Proliferation and Suppression Assays

The samples of three healthy adult donors were used in this assay. T cells from PBMCs were purified by positive selection using antibodies CD3 (Percp, Biolegend, USA) and CD4 (APC-CY7, BD) isolation *via* FCM. Purified CD4+ T lymphocytes were labeled with 5 μM 5,6-carboxy-fluorescein diacetate succinimidyl ester (CFSE) (eBioscience) and thereafter were cocultured with isolated MAIT cells to 4 × 10^4^ CFSE-labeled CD4+T cells at a ratio of 2:1, 1:1, 1:2, and 1:4 under the presence or absence of anti-CD3/CD28 beads (Thermo) in RPMI 1640 (Biological Industries) supplemented with 10% fetal bovine serum (FBS) in 96-well U-bottom plates. After 4 days, cells were harvested for FCM analysis.

### *In Vitro* Mucosa-Associated Invariant T Cell Activation and Cytokine Detection Assays

For the experiment, 25 transplant patients hospitalized in our institute from July 10, 2020, to October 1, 2020, were enrolled, including gut aGVHD onset (eight cases), fever or infections at the same period (10 cases), and no events at the same period (seven cases) (**Supplementary Table S2**). The sorted PBMCs from one sample were placed in two wells of flat-bottom plates, with 1 × 10^6^ cells in each well. One well was treated with anti-CD3/CD28 beads (Thermo) 1 μg/ml, incubated at 37°C, 5% CO_2_ for 2 h and was added with Golgisop 1 μl/ml, incubated at 37°C, 5% CO_2_ for 4 h. Another well was added with IL-12 100 ng/ml and IL-18 100 ng/ml, incubated overnight. After 20 h, the well was added with Golgisop 1 μl/ml and incubated at 37°C, 5% CO_2_ for 4 h. Finally, cells from the two wells were respectively collected and stained with antibodies following intracellular staining protocol for FCM analysis.

### Immunofluorescence

Intestinal tissues were obtained from two healthy donors and five gut aGVHD patients. Paraffin-embedded intestinal tissue samples were prepared for immunofluorescence. Sections were stained with primary antibody CD161 (1:400, Abcam, ab197979, UK) together with CD8 (1:400, Cell Signaling Technology, 70306S, USA). Biotinylated secondary antibody was performed using the EnVision+System-HRP (AEC) (K4005, Dako, Glostrup, Denmark) (Details in the **Supplementary Material and Methods**).

### Gut Microbiota Profiling

Stool samples were subjected to 16S rRNA V3-V4 region high-throughput sequencing to detect the abundance of intestinal flora. The sequencing was based on the IonS5™XL sequencing platform using the single-end sequencing (Single-End) method to construct a small fragment library for single-end sequencing (Details in the **Supplementary Material and Methods**).

### Transplantation Protocol

Granulocyte colony-stimulating factor (G-CSF, 5 mg/kg/day for 5 days) was used to mobilize the bone marrow (G-BM) and peripheral blood (G-PB). The target mononuclear cell count (MNC) was greater than 6 × 10^8^/kg. Unmanipulated BM (harvested on day 4 after G-CSF) and peripheral blood stem cells (PBSCs, harvested on day 5 after G-CSF) were infused into the recipient on the day of collection. All patients received both G-BM and G-PB or only G-PB as allografts.

All the patients in this study received myeloablative conditioning regimens (Details in the **Supplementary Material and Methods**).

### Acute Graft-Versus-Host Disease Prevention and Treatment Protocols

The diagnosis of gut aGVHD depends on histopathological diagnosis provided by rectal or colonoscopy. aGVHD was diagnosed and graded based on the established criteria (35, 36). aGVHD diagnosis was based on clinical features and pathological findings. The aGVHD prevention protocol is a cyclosporine (CSA)/methotrexate (MTX)/mycophenolate mofetil (MMF) combined protocol (Details in the **Supplementary Material and Methods**).

### Donor Lymphocyte Infusion

Prophylactic donor lymphocyte infusion (DLI) was administered for patients in relapse or no remission (NR) state before transplantation. The indications for DLI included hematological leukemia relapse, receiving chemotherapy followed by DLI, or positive Minimal residual disease (MRD) detection as previously described (37).

### Statistical Analysis

Patient variables were compared using the chi-square test for categorical variables. The distribution of continuous variables was calculated using the Mann–Whitney U-test. Cumulative incidences of aGVHD and relapse were estimated to accommodate competing risks with 95% confidence intervals. Comparisons between cumulative incidences were performed by the Gray test. The probabilities of overall survival (OS) and disease-free survival (DFS) were estimated with the Kaplan–Meier method and compared using the log-rank test. Multivariate analyses were performed using the Cox proportional hazards model for survival to identify the independent prognostic variables. The parameters with p < 0.1 according to the univariate analysis were entered into a multivariate model. To analyze the association between donor characteristics and graft cell composition, logistic regression analyses were conducted to determine the independent donor factors involved in donor dichotomous variables selected from the univariate analysis. Analyses were performed using GraphPad Prism 6.0 and SPSS version 23 software (Chicago, IL, USA). p-values <0.05 were considered statistically significant.

## Results

### Patient Characteristics

One hundred fifty transplanted patients, including 89 males and 61 females, were continuously followed, with a median age of 35 (14–63) years. For the infused grafts, 17 cases were infused with PBSCs only, and 133 cases were infused with PBSCs combined with BMSCs. There were 59 aGVHD cases after transplantation, including 25 grade I aGVHD and 34 grade II–IV aGVHD, of which 16 cases were gut aGVHD. The median days of gut aGVHD onset and complete remission (CR) were 43 (27–98) days and 65 (40–120) days after transplantation, respectively. Other characteristics are shown in **Supplementary Table 1**.

### The Occurrence of Gut Acute Graft-Versus-Host Disease Was Related to the Number of Mucosa-Associated Invariant T Cells in the Graft

**Figure 1:**
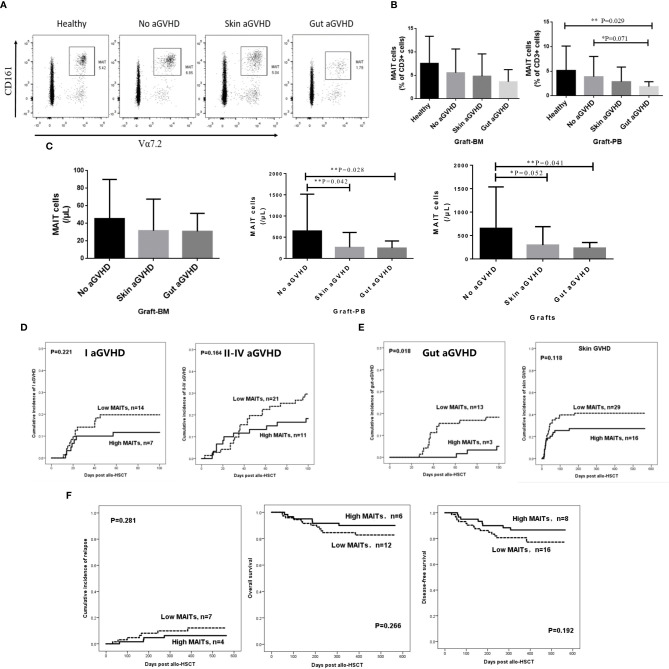
Comparison of the frequency and number of mucosa-associated invariant T (MAIT) cells in the grafts from the recombinant human granulocyte colony-stimulating factor (rhG-CSF) mobilized donors and healthy person. **(A)** Flow cytometry of MAIT cells in the grafts of four patient groups. **(B)** The frequency of MAIT cells (% of CD3+ cells) in graft-bone marrow (BM) [Healthy, n = 4; No acute graft-versus-host disease (aGVHD), n = 21; Skin aGVHD, n = 28; Gut aGVHD, n = 12] and graft-peripheral blood (PB) (Healthy, n = 8; No aGVHD, n = 24; Skin aGVHD, n = 27; Gut aGVHD, n = 15; Gut aGVHD *vs.* Healthy, p = 0.029). **(C)** The number of MAIT cells in graft-BM, graft-PB (Gut aGVHD *vs.* no aGVHD, p = 0.028; Skin aGVHD *vs.* no aGVHD, p = 0.042), and the total grafts (No aGVHD, n = 16; Skin aGVHD, n = 13; Gut aGVHD, n = 13). **(D)** The influence of the high MAIT (>5.3 × 10^6^/kg) and low MAIT (<5.3 × 10^6^/kg) group level in the graft on the occurrence of aGVHD (I aGVHD and II–IV aGVHD). **(E)** The influence of the high MAIT (>5.3 × 10^6^/kg) and low MAIT (<5.3×10^6^/kg) groups in infused graft on gut aGVHD (p = 0.018) or skin aGVHD. **(F)** The influence of the high MAIT (>5.3 × 10^6^/kg) and low MAIT (<5.3 × 10^6^/kg) groups in infused graft on the prognosis after transplantation. Levels of significances are given as p-values with **≤0.05 and *≤0.1.

To explore the relationship between the number of MAIT cells in infused grafts and the occurrence of gut aGVHD after transplantation, we divided the infused grafts into gut aGVHD, skin aGVHD, and no aGVHD groups based on the posttransplant events of recipients. The FCM definition of MAIT cells and the comparison of MAIT cells in FCM between the three groups and healthy individuals are shown in **Supplementary Figure 1A** and **Figure 1A**. The data showed that the frequency of MAIT cells in the gut aGVHD group was the lowest among the four groups. Especially in G-PB, the frequency of MAIT cells in the gut aGVHD group was obviously lower than that in the healthy group (gut aGVHD *vs.* healthy, p = 0.029; **Figure 1B**). In infused grafts, the number of MAIT cells in the gut aGVHD and skin aGVHD groups was lower than that in the no GVHD group (G-PB: Gut aGVHD *vs.* no aGVHD, p = 0.028; Skin aGVHD *vs.* no aGVHD, p = 0.042; Grafts: Gut aGVHD *vs.* no aGVHD, p = 0.041; **Figure 1C**). In addition, Tc and Treg cells related to the occurrence of aGVHD were analyzed, and the number and frequency of these two types of cells in infused grafts were not significantly different in these groups (**Supplementary Figures 1B–D**). **Supplementary Table 4** shows that the number of CD34+ cells in graft-BM was significantly different between the skin aGVHD and no aGVHD groups (p = 0.043). Furthermore, consistent with the results of **Figure 1**, the number of MAIT cells in infused grafts in patients with gut aGVHD was significantly lower than that in patients without GVHD (G-PB: p = 0.003; Grafts: p = 0.005; **Supplementary Table 4**). The other remaining factors, such as sex and weight of donors, Tc, and MNC, had no significant impact on the occurrence of aGVHD.

We further explored whether MAIT cells in the graft also play roles in other intestinal diseases after transplantation, such as posttransplant diarrhea caused by chemotherapy or infection. Thus, the grafts were divided into a gut aGVHD group (group 1) and a chemotherapy- or infection-induced diarrhea group (group 2). The characteristics of these two groups of patients are shown in **Supplementary Table 5**. The frequency of MAIT cells of group 1 was lower than that of group 2 in PBSC (PBSC: Group 1 *vs.* Group 2, p = 0.005; **Supplementary Figure 1E**). Furthermore, the number of MAIT cells of group 1 was lower than that of group 2 (p = 0.036; **Supplementary Figure 1E**).

The above results confirmed that the number of infused MAIT cells affected the occurrence of gut aGVHD. On this basis, we determined a median number of MAIT cells (5.3 × 10^6^/kg) in infused grafts to divide the 150 transplant patients into two groups with high and low numbers of MAIT cells in infused grafts (High MAITs >5.3 × 10^6^/kg; Low MAITs <5.3 × 10^6^/kg) and observed the incidence of aGVHD and prognosis between the two groups (**Figures 1D–F**). The results showed that the low MAIT group had a higher incidence of grade I aGVHD or grade II–IV aGVHD than that in the high MAIT group, although the difference was not significant (**Figure 1D**). Furthermore, the low MAIT group was more likely to develop gut aGVHD and skin aGVHD than the high MAIT group. Especially for gut aGVHD, there was a significant difference between the two groups (p = 0.018; **Figure 1E**). However, the cumulative incidence of relapse (CIR), OS, and DFS of the two groups were not obviously different (**Figure 1F**).

### Recipients With a High Number of Mucosa-Associated Invariant T Cells in Infused Grafts Had a Higher Abundance of Intestinal Flora After Transplantation

**Figure 2:**
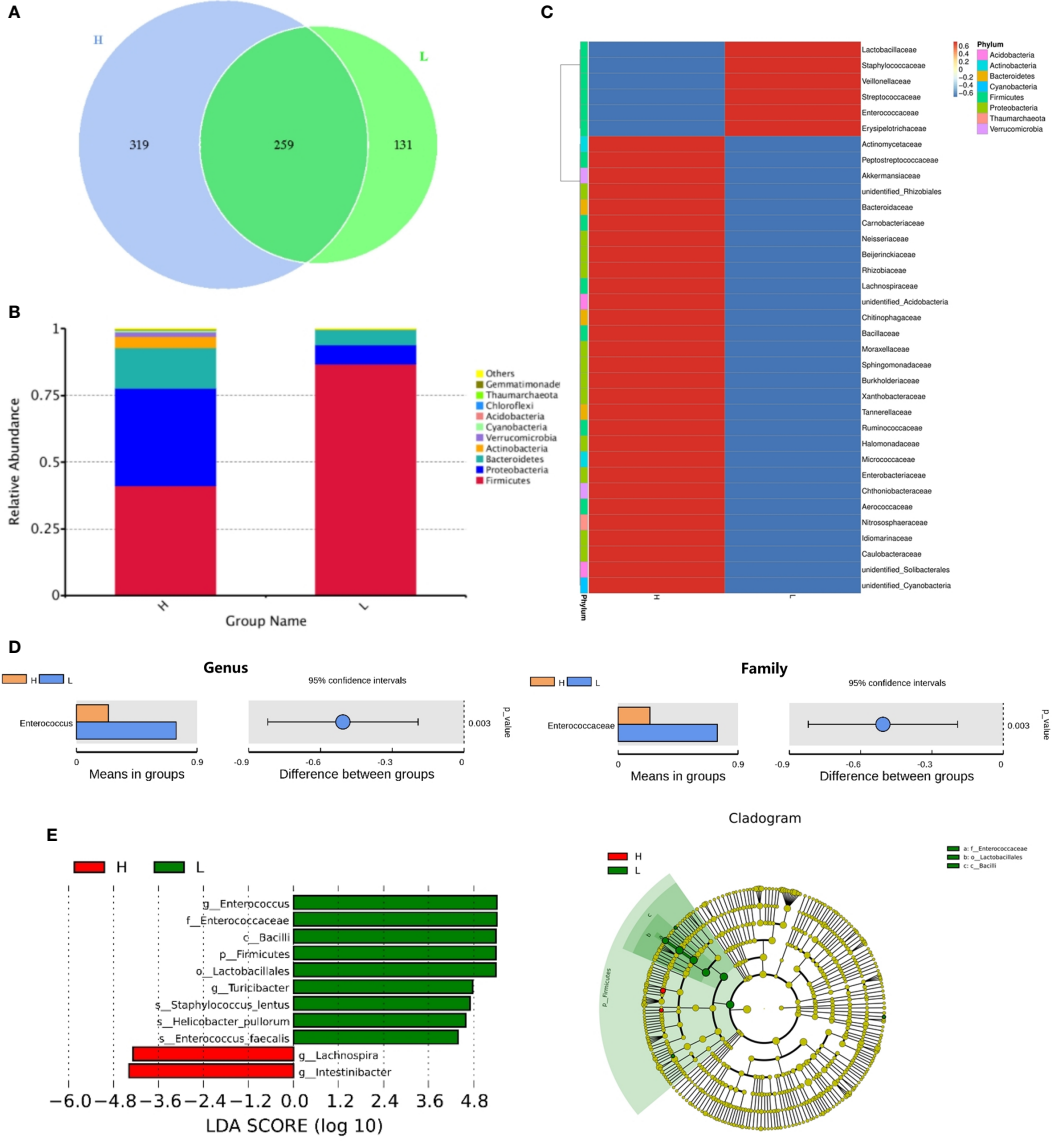
The comparison of the intestinal flora abundance of recipients on posttransplant 14 days between high number of mucosa-associated invariant T (MAIT) cells (>5.3 × 10^6^/kg, H group, n = 12) and low number of MAIT cells (<5.3 × 10^6^/kg, L group, n = 10) in infused grafts. **(A)** Venn diagram, each circle represents a group, the number in the overlapped part of the circle and circle represent the number of operational taxonomic units (OTUs) shared between the two groups, and the number without overlap represents the unique OTUs of the group. **(B)** The column chart of relative abundance of species at phylum level. The abscissa is the group name. The ordinate (Relative Abundance) represents the relative abundance. *Others* represents the sum of the relative abundances of all the phyla except these 10 phyla in the figure. **(C)** Cluster heat map of species abundance at the genus level. According to the species annotation and abundance information of all samples at the genus level, select the top 35 abundant genera. **(D)** T-test species difference analysis diagram at the genus and family levels between H and L groups. **(E)** Latent Dirichlet Allocation (LDA) score distribution histogram and cladogram. The LDA score distribution histogram shows the Biomarker species with statistical differences between the groups whose LDA Score is greater than the set value (the default setting is 4). The length of the histogram represents the impact of different species (that is, LDA Score). In the cladogram, the circles radiating from the inside to the outside represent the taxonomic level from the phylum to the genus (or species).

Next, we investigated whether the MAIT cell number in infused grafts would affect the intestinal flora abundance of posttransplant recipients. **Figure 2** showed the comparison of the intestinal flora abundance of recipients at day +14 between groups with high and low MAIT cells in infused grafts. As expected, the High MAIT group had more intestinal flora species and more abundant *Bacteroidetes*, *Proteobacteria*, and *Actinobacteria* at +14 days than those in the Low MAIT group (**Figures 2A, B**). At the genus level, the intestinal flora of the High MAIT group at +14 days was rich in a variety of beneficial bacteria, such as *Blautia*, *Lachnoclostridium*, and *Faecalibacterium*, and that of the Low MAIT group was rich in *Enterococcus*, *Streptococcus*, and *Lactobacillus* (**Figure 2C**). Furthermore, species difference analysis found that the Low MAIT group had a higher abundance of *Enterococcus* than the High MAIT group at the genus (p = 0.003) and family (p = 0.003) levels (**Figures 2D, E**). Additionally, a higher abundance of *Lactobacillales* was found in the Low MAIT group than in the High MAIT group at the order level (p = 0.005; **Supplementary Figure 2**). The Low MAIT group had a higher abundance of *Firmicutes* (p = 0.002) and a lower abundance of *Proteobacteria* (p = 0.023) than the High MAIT group at the phylum level (**Supplementary Figure 2**). In summary, these results suggested that the number of MAIT cells in infused grafts may affect the abundance of intestinal flora in the early posttransplant period.

### Reconstitution of Mucosa-Associated Invariant T Cells and Their Relationship With Gut Acute Graft-Versus-Host Disease

**Figure 3:**
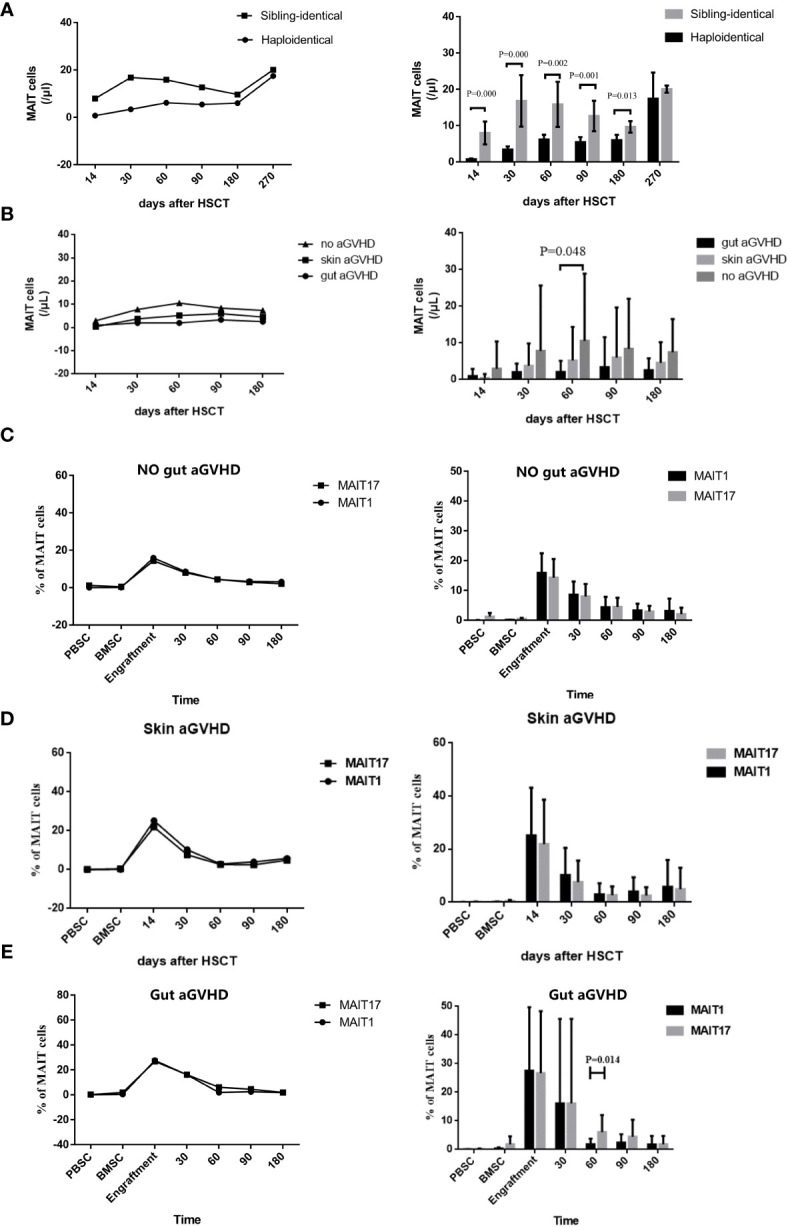
Reconstitution of mucosa-associated invariant T (MAIT) cells and their different subgroups in the peripheral blood after transplantation. **(A)** Posttransplant number of MAIT cells reconstituted under haplo-hematopoietic stem cell transplantation (HSCT) and sibling-identical HSCT. **(B)** Posttransplant number of MAIT cells reconstituted in gut acute graft-versus-host disease (aGVHD) patients, skin aGVHD and no gut aGVHD patients. **(C)** Posttransplant reconstitution of MAIT1 and MAIT17 subsets based on the expression of transcription factors T-bet and Rorgt respectively in no gut aGVHD patients. **(D)** Posttransplant reconstitution of MAIT1 and MAIT17 subsets based on the expression of transcription factors T-bet and Rorgt respectively in skin aGVHD patients. **(E)** Posttransplant reconstitution of MAIT1 and MAIT17 subsets based on the expression of transcription factors T-bet and Rorgt respectively in gut aGVHD patients.

Studying the MAIT cell reconstitution after transplantation is conducive to better analysis of the functional role of MAIT cells in different disease states in the transplantation system. The results showed that MAIT cells in PB increased rapidly within 30 days after both haplo-HSCT and sibling-identical HSCT, which was similar to the reconstitution observed in previous reports (29, 30, 38). Within +180 days, the number of MAIT cells in haplo-HSCT patients was significantly lower than that in sibling-identical HSCT patients (p < 0.05; **Figure 3A**), and the difference in reconstitution between the two groups gradually decreased after +180 days. However, the frequency of MAIT cells in both transplant types was at a normal level, and there was no significant difference (**Supplementary Figure 3A**). We then observed that within +180 days, the gut aGVHD, skin aGVHD, and no aGVHD groups showed that MAIT cells constituted in decreasing order, and there is a statistical difference at +60 days between gut aGVHD and no aGVHD (p = 0.048; **Figure 3B**). Furthermore, the frequency of MAIT cells in patients with gut aGVHD tended to increase after +60 days, but it was not statistically significant (**Supplementary Figure 3B**).

We next observed the reconstitution of subsets MAIT1 and MAIT17. The results showed that in the skin aGVHD and no gut aGVHD groups, the reconstitution was not significantly different between MAIT1 and MAIT17 (**Figures 3C, D**). However, in gut aGVHD patients, the proportion of MAIT17 (p = 0.014; **Figure 3E**) significantly increased at +60 days, which suggested that MAIT17 may play an important functional role at the onset of gut aGVHD.

### Effects of Mucosa-Associated Invariant T Cells on Gut Acute Graft-Versus-Host Disease Under Different Stimulation Signals

**Figure 4:**
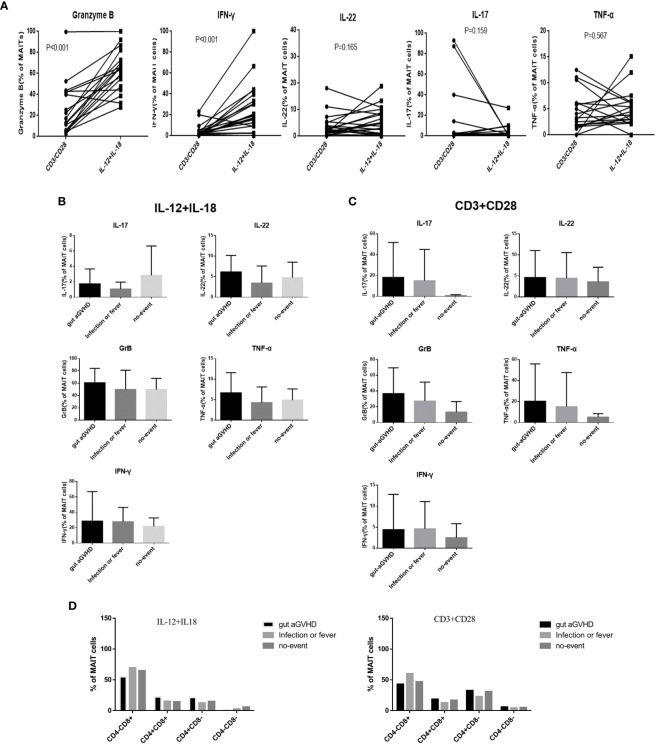
The comparison of the cytokine expression of mucosa-associated invariant T (MAIT) cells in peripheral blood under different stimulation signals *in vitro* among the gut acute graft-versus-host disease (aGVHD) group (n = 8), infection or fever group (n = 10), and no-event group (n = 7). **(A)** The comparison of the cytokine expression of MAIT cells under different stimulation signals *in vitro*. **(B)** The comparison of the cytokine expression of MAIT cells under interleukin (IL)-12/IL18 stimulation *in vitro* among the gut aGVHD group (n = 8), infection or fever group (n = 10), and no-event group (n = 7). **(C)** The comparison of the cytokine expression of MAIT cells under cluster of differentiation (CD)3/CD28 stimulation signals *in vitro* among the gut aGVHD group (n = 8), infection or fever group (n = 10), and no-event group (n = 7). **(D)** The comparison of cytokine expression under IL-12/IL18 stimulation and CD3/CD28 stimulation.

The CD3/CD28 antibody can mimic the dual signal of T-cell activation and act through TCR (TCR-dependent pathway). IL-12/IL-18 receptor is highly expressed on the surface of MAIT cells and can mimic the cytokine activation pathway (non-TCR-dependent pathway). We used CD3/CD28 and IL-12/IL-18 to respectively stimulate the sorted MAIT cells in PB. The data analysis showed that MAIT cells secreted more GrB and IFN-γ under IL-12/IL-18 than CD3/CD28 stimulation (p < 0.001; **Supplementary Figures 4A, B** and **Figure 4A**), while CD3/CD28 stimulation caused MAIT cells to express more IL-17 (**Figure 4A**). Furthermore, IL-22, GrB, TNF-α, and IFN-γ tended to increase under IL-12/IL-18 stimulation at the onset of gut aGVHD (**Figure 4B**), while IL-22, IL-17, GrB, TNF-α, and IFN-γ had an increased tendency under CD3/CD28 stimulation at the onset of gut aGVHD (**Figure 4C**). The above data suggested that the expression of IL-17 may occur mainly through CD3/CD28 (TCR-dependent) pathways. In addition, the expression of MAIT cell proliferation marker Ki67 under the stimulation of CD3/CD28 and IL-12/18 tended to increase than that of under no stimulation (**Supplementary Figure 4C**). For changes in each MAIT cell subset, the data showed a significant change: under CD3/CD28 stimulation, CD8+ MAIT cells were decreased and CD4-CD8- MAIT cells in the gut aGVHD group were increased compared with IL-12/IL-18 (**Figure 4D**). Furthermore, in the comparison of the cytokines of various subsets, almost all IL-17 was secreted by CD4-CD8- MAIT cells under IL-12/IL-18 stimulation (**Supplementary Figure 4D**). The above section still lacks statistically significant data to further support these results. A larger amount of data or *in vitro* experiments will still be needed for further verification.

### Changes in Mucosa-Associated Invariant T Cells and Intestinal Flora in Patients With Gut Acute Graft-Versus-Host Disease Around the Onset of Gut Acute Graft-Versus-Host Disease

**Figure 5:**
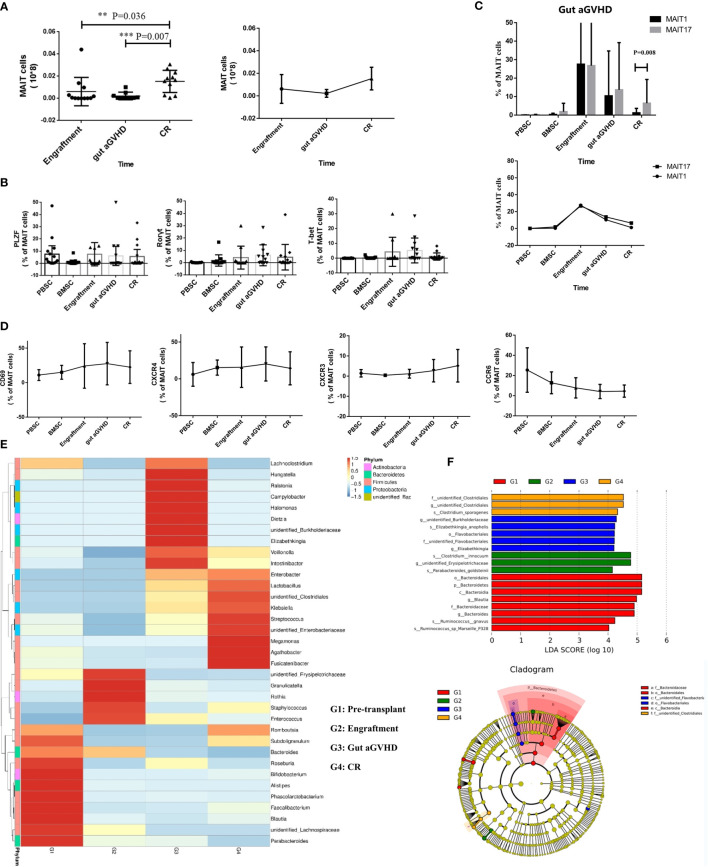
Mucosa-associated invariant T (MAIT) cell functional changes in 16 gut acute graft-versus-host disease (aGVHD) patients before and after the onset of gut aGVHD. **(A)** The changes of MAIT cell number at three posttransplant time points, namely, at the time of neutrophil engraftment, at the onset of gut aGVHD, and at the CR of gut aGVHD. **(B)** The changes of transcription factors PLZF, T-bet, and Rorγt in patients with gut aGVHD at different points. **(C)** Posttransplant comparison of MAIT1 and MAIT17 subsets based on the expression of transcription factors T-bet and Rorγt respectively in gut aGVHD patients. **(D)** The changes of activation markers and chemokine receptors in patients with gut aGVHD at different points. **(E)** Cluster heat map of species abundance at the genus level. **(F)** LDA score distribution histogram and cladogram. Levels of significances are given as p-values with ***≤0.01 and **≤0.5.

We compared the number of MAIT cells in PB of 16 patients with gut aGVHD at three time points after transplantation [the time of neutrophil implantation, at the onset of gut aGVHD, and at the complete response (CR) of gut aGVHD]. As shown in the *Results*, the number of MAIT cells at the onset of gut aGVHD in PB was significantly lower than that at the engraftment and CR points (engraftment *vs.* gut aGVHD, p = 0.036; CR *vs.* gut aGVHD, p = 0.007; **Figure 5A**). We also defined a group of non-MAIT cells (CD3+CD161-Vα7.2+) as a control and observed that the number of non-MAIT cells did not show changes similar to those of MAIT cells at the three time points (**Supplementary Figure 5A**).

The expression of promyelocytic leukaemiazinc finger (PLZF), T-bet, and Rorγt is related to the maturity and function of MAIT cells (18, 25, 28). The data showed that T-bet and Rorγt had an increasing trend at the onset of gut aGVHD, but PLZF did not demonstrate this trend (**Figure 5B** and **Supplementary Figure 5B**). **Figure 5C** further supported the above results and suggested that MAIT cells transformed into mature subsets MAIT1 and MAIT17 after gut aGVHD onset. In addition, the activation marker CD69 and CXCR4 showed an increasing trend at the onset of gut aGVHD (**Figure 5D**). For MAIT cell subsets, we found that CD4-CD8- MAIT cells and CD4-CD8+ MAIT cells expressed most of the activation markers and chemokine receptors, which suggested that the two subsets could be more mature than the other single positive subsets, especially CD4-CD8- MAIT cells. CD4+CD8+ MAIT cells and CD4-CD8- MAIT cells expressed most transcription factors, and CD4+CD8+ MAIT cells expressed more PLZF and Rorγt, while CD4-CD8- MAIT cells expressed more T-bet and Rorγt at the onset of gut aGVHD (**Supplementary Figure 5C**).

Correspondingly, the results for the intestinal flora showed that at the onset of gut aGVHD, *Firmicutes* was significantly reduced, and there was normal intestinal rare flora such as *Ralstonia* and *Campylobacter* compared with the other three points (**Figure 5E** and **Supplementary Figure 5D**). Furthermore, significant difference analysis showed that different levels of *Flavobacteriales* were predictive flora at the onset of gut aGVHD (**Figure 5F**).

### Comparison of Mucosa-Associated Invariant T Cells and Intestinal Flora Among Patients With Gut Acute Graft-Versus-Host Disease and Other Events

**Figure 6:**
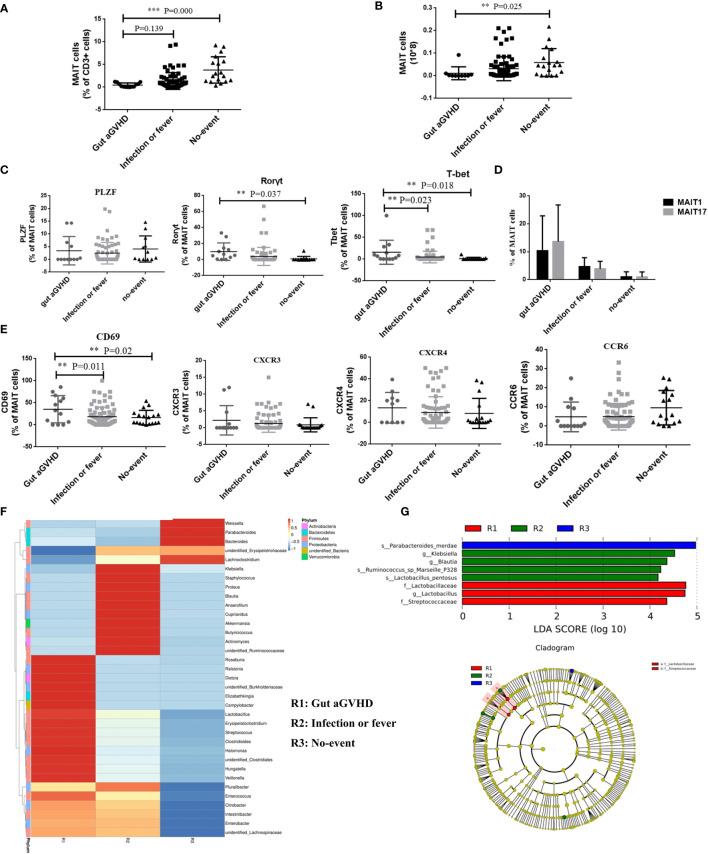
The comparison of the number and function of mucosa-associated invariant T (MAIT) cells among patients with gut acute graft-versus-host disease (aGVHD) (n = 16), patients with infection or fever (n = 38) at the same time, and patients with no-event (n = 19) at the same time. **(A)** The comparison of the frequency of MAIT cells among gut aGVHD group, infection or fever group, and no-event group (Frequency: Gut aGVHD *vs.* No-event, p < 0.001). **(B)** The comparison of the number of MAIT cells among gut aGVHD group, infection or fever group, and no-event group (Number: Gut aGVHD *vs.* No-event, p = 0.025). **(C)** The comparison of T-bet+ MAIT (MAIT1) and Rorγt+ MAIT (MAIT17) among the three groups. **(D)** The comparison of the transcription factors among the three groups (Rorγt: Gut aGVHD *vs.* No-event, p = 0.037; T-bet: Gut aGVHD *vs.* No-event, p = 0.018; Gut aGVHD *vs.* Infection or fever, p = 0.023). **(E)** The comparison of the chemokine factors and markers among the three groups (Gut aGVHD *vs.* No-event, p = 0.02; Gut aGVHD *vs.* Infection or fever, p = 0.011). **(F)** Cluster heat map of species abundance at the genus level. **(G)** LDA score distribution histogram and cladogram. Levels of significances are given as p-values with ***≤0.01 and **≤0.5.

We further compared the changes in the number and function of MAIT cells among the three groups of patients with different disease statuses during the same posttransplant period. The results showed that compared with the infection or fever group and the no-event group, the frequency and number of MAIT cells at the onset of gut aGVHD were significantly decreased, and the difference was significant between the gut aGVHD and no-event groups (frequency: gut aGVHD *vs.* no-event, p < 0.001; number: gut aGVHD *vs.* no-event, p = 0.025; **Figures 6A, B** and **Supplementary Figure 6A**). Furthermore, compared with the infection or fever and no-event groups, MAIT cells expressed more transcription factors Rorγt (Rorγt: Gut aGVHD *vs.* No-event, p = 0.037; **Figure 6C** and **Supplementary Figure 6B**) and T-bet (T-bet: Gut aGVHD *vs.* No-event, p = 0.018; Gut aGVHD *vs.* Infection or fever, p = 0.023; **Figure 6C** and **Supplementary Figure 6B**), and the frequency of MAIT17 was significantly higher than that of MAIT1 at the onset of gut aGVHD (**Figure 6D**). Furthermore, the proportion of CD4-CD8-MAIT cells at the onset of gut aGVHD was obviously increased compared with that in the other two groups (**Supplementary Figure 6C**). The data also showed that MAIT cells expressed more CD69 (gut aGVHD *vs.* no-event, p = 0.02; gut aGVHD *vs.* infection or fever, p = 0.011; **Figure 6E**), CXCR3, and CXCR4 in the gut aGVHD group than in the other two groups. The analysis of intestinal flora found that the abundance of flora (*Veillonella*, *Enterococcus*) associated with the high incidence of GVHD at the onset of gut aGVHD was higher than that in the other two groups (**Figure 6F**). Furthermore, *Lactobacillus* could be a predictive flora of the gut aGVHD group compared with the other two groups (**Figure 6G**).

### Mucosa-Associated Invariant T Cells Inhibit the Proliferation of CD4+ T Cells *In Vitro*

**Figure 7:**
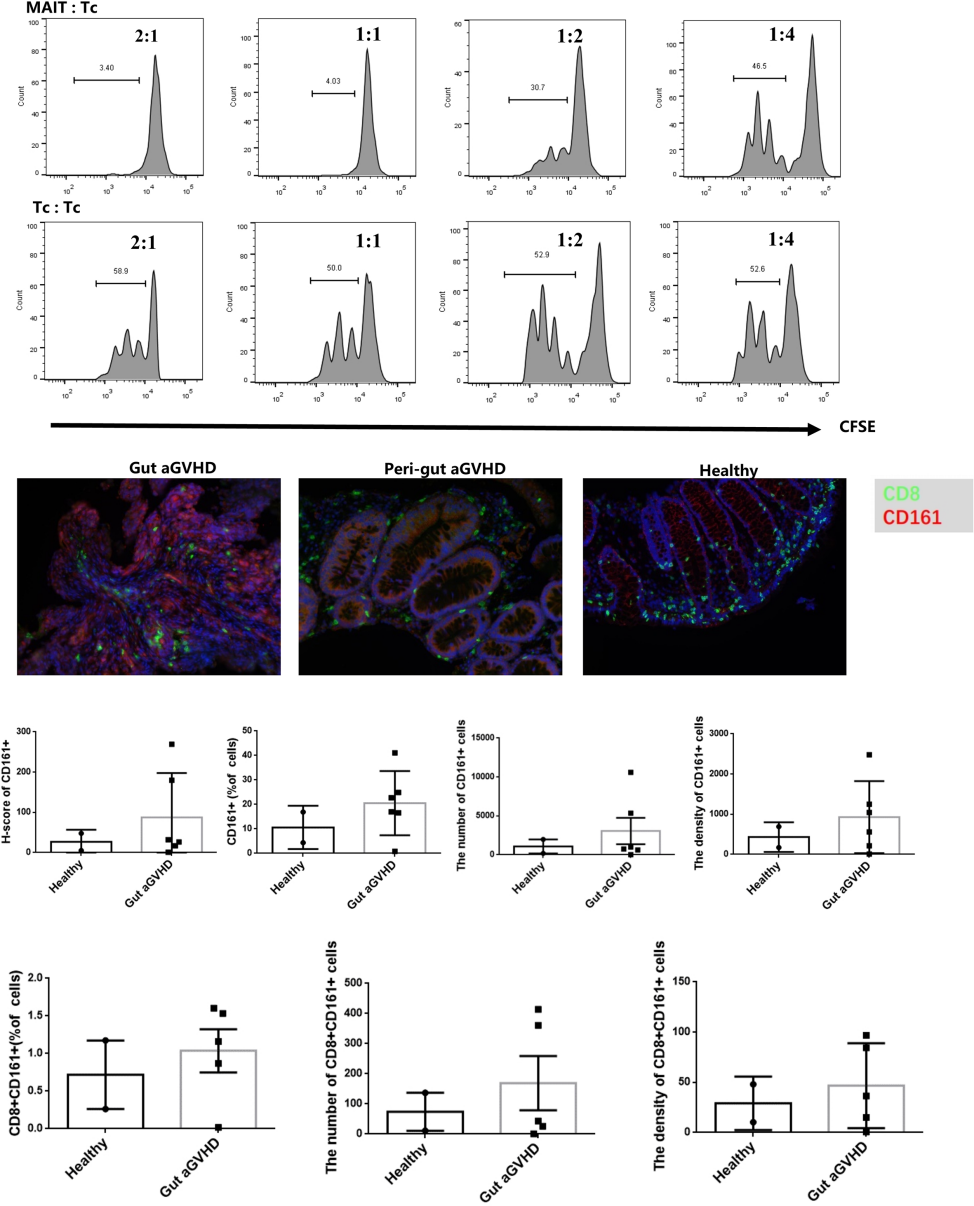
The *in vitro* inhibition experiment of mucosa-associated invariant T (MAIT) cells on CD4+ T cells and immunofluorescence experiment of intestinal tissue. **(A)** MAIT cells and CD4+ T cells were isolated from the peripheral blood of four healthy donors. Here, 5,6-carboxy-fluorescein diacetate succinimidyl ester (CFSE)-labeled CD4+ T cells with CD3/CD28 bead stimulation and MAIT cells or CD4+ T cells were mixed and cultured at a ratio of 2:1, 1:2, 1:4, and 1:8 for 4 days. Representative flow cytometric analysis of CFSE dilutions in CD4+ T cells. **(B)** Immunofluorescence of intestinal tissues of healthy people, gut aGVHD lesion sites and peri-gut aGVHD sites of gut aGVHD patients. **(C)** Comparison of CD161 staining intensity, CD161-positive cell ratio, number, and density between healthy person intestinal tissue (n = 2) and intestinal lesion tissue of gut aGVHD patients (n = 5). Since only one case of gut aGVHD patient in the experiment received the intestinal tissue of peri-gut aGVHD site, there is no peri-gut aGVHD group in the comparison. **(D)** Comparison of CD8+CD161+ cell ratio, number, and density between healthy person intestinal tissue (n = 2) and intestinal lesion tissue of gut aGVHD patients (n = 5).

Next, we would prove whether MAIT cells could affect the occurrence of gut aGVHD through immunosuppressive effects. The results showed that with a higher proportion of MAIT cells in mixed culture, the inhibitory effect of MAIT cells on CD4+ T cells was stronger (**Figure 7A** and **Supplementary Figure 7A**), while this result did not appear in the control group without CD3/CD28 stimulation (**Supplementary Figures 7B, C**). This suggested that MAIT cells may have a certain immunosuppressive effect on the proliferation of CD4+ T cells *in vitro*.

### Increase in CD8+CD161+ Cells in Lesions of the Intestinal Tract During Gut Acute Graft-Versus-Host Disease

When infection or inflammation occurs, the MAIT cells in PB may be chemoattracted to lesion sites under the action of chemokines (22–26). Therefore, the number of MAIT cells in PB may decrease at this time, while MAIT cells in lesion sites may increase. In this study, we initially used CD161 and Vα7.2 antibodies to define MAIT cells in intestinal tissues, but Vα7.2 staining failed in the preliminary experiment. Previous studies have confirmed that more than 90% of CD8+CD161^hi^ cells are MAIT cells (33, 34). Therefore, we switched to CD8 and CD161 antibodies for staining. Our results showed that compared with healthy donors and perigut aGVHD (non-diseased tissue around the gut aGVHD lesion in the intestinal tract) (**Figure 7B**), glands in the lesions were extensively destroyed when gut aGVHD occurred. The ratio, number, and density of CD161+ and CD8+CD161+ cells increased (**Figures 7C, D**), while CD8+ and CD8+CD161- cells showed no obvious change or decreased (**Figure S8**). Noteworthy, due to the limited number of intestinal tissue samples, this result needs to be further verified in a larger sample size or in a mouse model.

### Univariate and Multivariate Analyses of Multiple Factors That May Affect Acute Graft-Versus-Host Disease and Gut Acute Graft-Versus-Host Disease

For the occurrence of aGVHD, the analysis found that MAIT cell count in grafts was an independent risk factor (**Supplementary Table 6**). Multivariate analysis found that ABO-matched grafts (p = 0.046) and MAIT cell counts in infused grafts (p = 0.01) were independent risk factors for gut aGVHD (**Table 1**).

**Table 1:** Univariate and multivariate analyses of risk factors for the occurrence of gut aGVHD.

Characteristics	Univariate analyses			Multivariate analyses		
	HR	95% CI	p-value	HR	95% CI	p-value
Donor gender	0.400	0.114–1.405	0.153			
Donor ages	1.018	0.984–1.054	0.296			
Recipient ages	0.998	0.961–1.036	0.919			
Recipient gender	2.563	0.931–7.053	**0.068**	2.850	1.001–8.108	0.050
ABO-matched grafts	3.520	1.223–10.133	**0.020**	2.992	1.022–8.762	**0.046**
disease status (pretransplant CR *vs.* NR)	0.043	0–61.587	0.395			
Transplant types	0.733	0.209–2.571	0.627			
Donor-recipient gender match	0.923	0.496–1.717	0.799			
Conditioning regimen	0.973	0.480–1.972	0.939			
G-BM CD34+ counts	2.446	0.579–10.335	0.224			
G-BM Treg cell counts	8.126	0.066–999.075	0.393			
G-BM Tc cell counts	1.088	0.981–1.206	0.111			
G-BM MAIT cell counts	1.180	0.557–2.501	0.665			
G-PB CD34+ counts	1.008	0.743–1.367	0.961			
G-PB Treg cell counts	1.110	0.909–1.356	0.307			
G-PB Tc cell counts	1.004	0.996–1.011	0.337			
G-PB MAIT cell counts	0.906	0.801–1.025	0.116			
Graft CD34+ counts	0.988	0.777–1.256	0.919			
Graft Treg cell counts	1.114	0.913–1.358	0.288			
Graft Tc cell counts	1.004	0.996–1.011	0.321			
Graft CD4/CD8	1.226	0.688–2.186	0.489			
Graft MNC	0.983	0.760–1.270	0.893			
Graft MAIT cell counts ≥5.3 × 10^6^/kg (median value) *vs.* <5.3 × 10^6^/kg	0.174	0.039–0.772	**0.021**	0.137	0.030–0.620	**0.010**
Day +30 MAIT cell counts ≥1.1/μl (median value) *vs.* <1.1/μl	0.670	0.239–1.884	0.448			
Day +60 MAIT cell counts ≥2.9/μl (median value) *vs.* <2.9/μl	0.387	0.121–1.235	0.109			

The factors with p < 0.1 in univariate analysis were included in multivariate analysis.

P < 0.05 was marked in bold.

G-BM, Graft bone marrow; G-PB, Graft peripheral blood.

## Discussion

Based on the basic biological characteristics of MAIT cells, including their enrichment and distribution in the intestine, the expression of intestinal mucosal protective factors (such as IL-17), and activation by the riboflavin metabolites of intestinal flora, we hypothesized that MAIT cells could play an important role in the occurrence of gut aGVHD in human HSCT. In this study, we confirmed that in humans, MAIT cells may affect the occurrence of gut aGVHD by regulating the composition of intestinal flora and by exerting an immunosuppressive effect *via* suppressing T-cell proliferation.

The absolute number of MAIT cells in infused grafts can influence the reconstitution of MAIT cells after transplantation (29–32). From this, we inferred that the number of MAIT cells in infused grafts may affect the occurrence of gut aGVHD. The multiple sets of data in our study verified this inference that a high MAIT cell count in infused grafts was related to the low incidence of gut aGVHD. Previous reports have confirmed that the intestinal flora can affect the occurrence of GVHD through its metabolites (butyrate or lactase) (8, 9). Intestinal low butyrate and low lactase are related to the occurrence of GVHD (9). Interestingly, our data confirmed that recipients with low MAIT cells in infused grafts had a higher abundance of *Enterococcus* in the early posttransplant period, and it was previously proven that the dominance of *Enterococcus* (low lactase and low butyrate) in the early posttransplant period (days 0 to +21) is related to GVHD (9, 10, 29). Our result was the first confirmation that the number of MAIT cells in infused grafts can affect the abundance and components of intestinal flora in the early posttransplant period. The possible reasons for this result are as follows. First, the number of infused MAIT cells determines the MAIT cell number of recipients in the early posttransplant period (29–32). Second, the rapid reconstitution of MAIT cells after transplantation is related to the increase in the abundance of intestinal flora (such as *Blautia* and *Bifidobacterium*) (29, 32, 39–41), which may be due to the destruction of the intestinal mucosal barrier by pretransplant pretreatment with cytotoxic drugs, resulting in increased permeability of the intestinal epithelium that allows intestinal bacterial antigens to contact and activate (by MR1/TCR-dependent pathway) MAIT cells from grafts. This may also be the reason for the rapid proliferation of MAIT cells within +30 days. Finally, in the early posttransplant period, the MAIT cells from grafts and the proliferated MAIT cells posttransplantation may regulate the intestinal flora through immune function.

IL-17 has been confirmed to play an important role in maintaining the integrity of the intestinal mucosa (1, 12). From the above results, we inferred that the influence of intestinal flora on gut aGVHD mainly involved the activation of MAIT cells through its riboflavin metabolic pathway (MR1 pathway), and then the activated MAIT cells can resist the occurrence of gut aGVHD by secreting IL-17. Therefore, it is necessary to investigate whether the intestinal flora related to the occurrence of gut aGVHD in our results is the flora of the riboflavin metabolism pathway. We screened out flora related to the occurrence of gut aGVHD, including *Enterococcus*, *Streptococcus*, *Flavobacteriales*, *Lactobacillus*, and *Firmicutes.* Combined with a previous report (20), *Enterococcus*, *Streptococcus*, and *Lactobacillus* were impaired in riboflavin biosynthesis. *Bacteroidetes*, *Proteobacteria*, *Actinobacteria*, and *Firmicutes* have recently been shown to activate MAIT cells in decreasing order (21, 42). Thus, the results suggested that the high abundance of intestinal flora without the riboflavin metabolic pathway might promote the occurrence of gut aGVHD. We believe that the reason for this result is that, on the one hand, these non-riboflavin metabolic pathways in the intestinal flora cannot activate intestinal MAIT cells so that intestinal protective cytokines or barriers against inflammation are reduced, leading to the occurrence of gut aGVHD. On the other hand, the loss of the riboflavin biosynthesis pathway allows these bacteria to escape host detection mediated by MAIT cells and enhances pathogenicity (20). Of course, we hope that, in the future, there will be *in vitro* or mouse model studies to further confirm that these specific bacteria screened out in our study may affect the occurrence of gut aGVHD by affecting MAIT cells.

The transcription factor PLZF has been shown to play an important role in differentiation into functional MAIT cells with a memory phenotype, which may be the reason why MAIT cells can respond quickly to cytokines or TCR signals (15, 43). The development of MAIT17 is strongly dependent on TCR signaling (18, 44, 45). This may also explain why when gut aGVHD occurred in our study, the proportion of IL-17+ MAIT cells was higher under CD3/CD28 (TCR-dependent pathways) than under IL-12/IL-18 stimulation. At present, the biological role of CD4 and CD8 in MAIT cells is unclear, but there is evidence that CD4-CD8- MAIT cells may be derived from activated CD8+ MAIT cells *in vivo* and are more mature than CD8+ MAIT cells (15, 26). This is also consistent with our results; when gut aGVHD occurred, the overall proportion of CD8+ MAIT cells was reduced. In addition, the ratio of CD8+ MAIT cells stimulated by IL-12/IL-18 was generally higher than that of CD3/CD28. This is likely because the surface of CD8+ MAIT cells expresses higher levels of IL-12 and IL-18 receptors (26). However, the ratio of CD4-CD8- MAIT cells was higher under CD3/CD28 stimulation than under IL-12/IL-18 stimulation, and most IL-17 was secreted by CD4-CD8- MAIT cells. Therefore, we speculated that the activation of MAIT cells by MR1/TCR-dependent pathways could promote MAIT cells to transform into CD4-CD8- MAIT/MAIT17, and CD4-CD8- MAIT/MAIT17 could play the role of anti-gut aGVHD by secreting IL-17. These results showed that IL-12/IL-18 dominated the expression of cytotoxic factors, such as IFN-γ and GrB, while the MR1/TCR-dependent pathway was related to IL-17 expression. Furthermore, activated MAIT cells express chemokine receptors CXCR3 and CXCR4 (involved in trafficking to the intestine) (15, 46–48), which may chemoattract MAIT cells from PB or non-pathological sites to the pathological site. This might be why in our study, the number of MAIT cells decreased in PB and increased in gut aGVHD lesion tissue at the onset of gut aGVHD.

Taken together, the MR1/TCR-dependent pathway could mainly promote the development of MAIT cells and exert an anti-gut aGVHD effect, while IL-12/IL-18 and other cytokine signals mainly promote mature subsets to express more cytotoxic cytokines and exert an important graft-versus-leukemia (GVL) effect, thereby balancing the effects of anti-gut aGVHD and graft-versus-leukemia (GVL) to maintain immune homeostasis. Notably, the infused MAIT cell number had no significant effect on posttransplant relapse or overall prognosis. In addition to the explanation of the effects of certain preventive interventions after transplantation, even more important may be that MAIT cells have a certain GVL or antitumor effect. In previous studies *in vitro*, it has been observed that MAIT cells isolated from PB of healthy donors not only have lymphokine-activated killing activity but also have direct cytotoxic effects on chronic myeloid leukemia-K562 cell line through GrB and perforin (49). However, the current research on the GVL or antitumor effect of MAIT cells in hematological malignancies is limited, and the clinical prognosis of transplant patients is also related to many factors, such as the risk stratification, transplant type, and so on. Therefore, the GVL or antitumor effect of MAIT cells needs to be further verified in a single background model.

In summary, with all of the above results combined with previous reports, we have confirmed for the first time the relationship between MAIT cells, intestinal flora, and gut aGVHD in the human body (**Table 2**). MAIT cells are rapidly activated and proliferate under stimulation of the intestinal flora (MR1/TCR-dependent pathway) and cytokines (non-TCR-dependent pathway). Activated MAIT cells in turn can regulate the intestinal flora through immune effects. At the same time, activated MAIT cells can inhibit the occurrence of gut aGVHD by exerting immunosuppressive effects or expressing the intestinal mucosal protective cytokine IL-17. Our study may provide new clinical prevention and treatment strategies for gut aGVHD, including MAIT cell therapy or specific flora transplantation, and ultimately further improve the efficacy of transplantation.

**Table 2:** The relationship between MAIT cells, intestinal flora, and gut aGVHD in the human body.

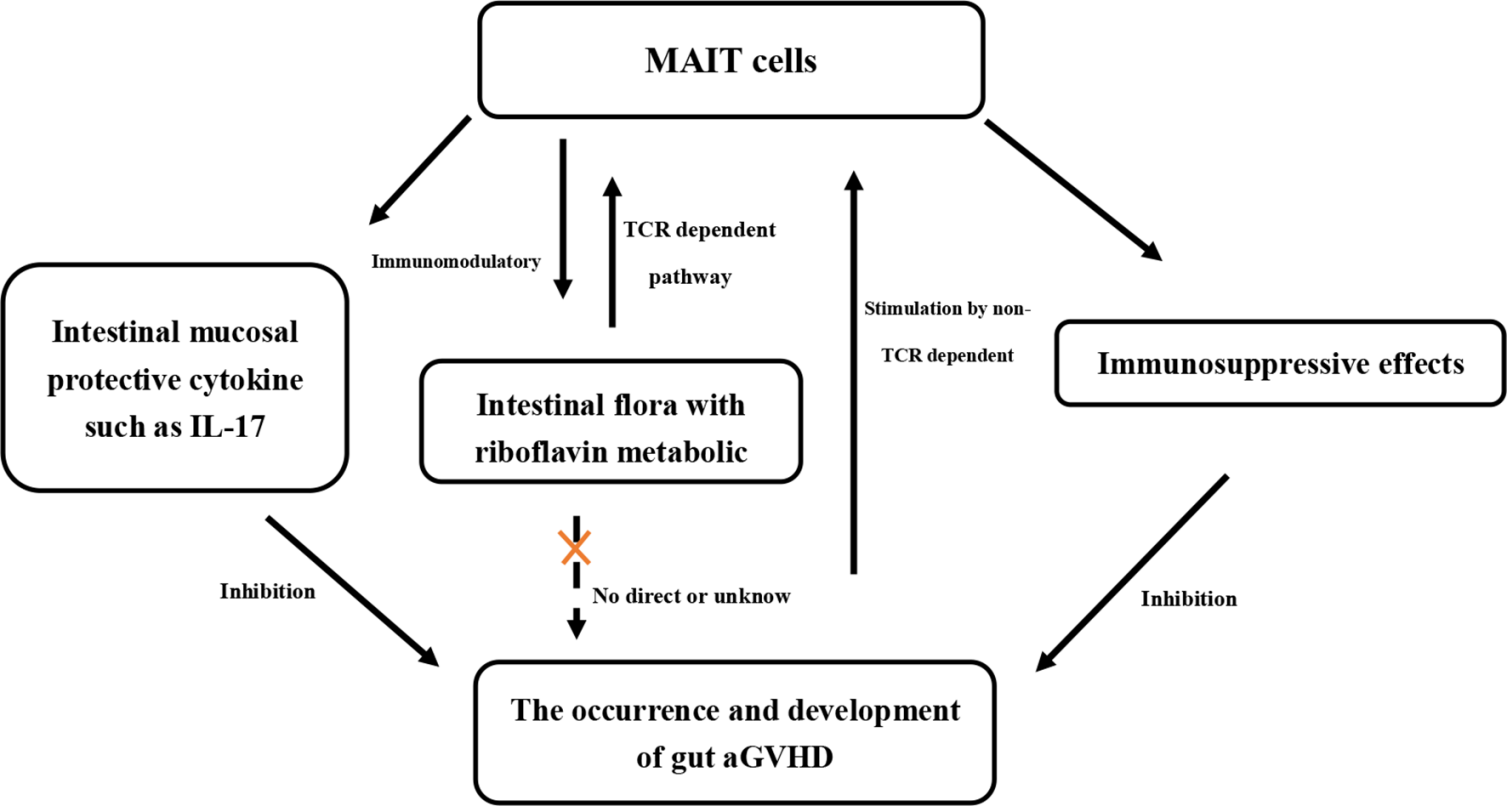

## Data Availability Statement

The original contributions presented in the study are included in the article/**Supplementary Material**. Further inquiries can be directed to the corresponding author.

## Ethics Statement

The study was approved by the Ethics Committee of Peking University People’s Hospital (Ethics number 2018PHB222-01). Written informed consent to participate in this study was provided by the participants’ legal guardian/next of kin. Written informed consent was obtained from the individual(s), and minor(s)’ legal guardian/next of kin, for the publication of any potentially identifiable images or data included in this article.

## Author Contributions

M-GG carried out the main experiments, analyzed the statistical analysis, and drafted the article. YH provided some experimental guidance and specimen collection. X-SZ designed the study, guided the experiments, and revised the article. X-YZ, Y-QS, JK, Z-DW, J-ZW, C-HY, YW, X-AP, and X-JH participated in the study to care for the critical patients. All authors contributed to the article and approved the submitted version.

## Funding

This work was supported by the National Key Research and Development Program of China (2017YFA0104500), the National Natural Science Foundation of China (grant no. 81870137), the Innovative Research Groups of the National Natural Science Foundation of China (grant no. 81621001), and the Beijing Municipal Science and Technology Commission (Z181100009618032).

## Conflict of Interest

The authors declare that the research was conducted in the absence of any commercial or financial relationships that could be construed as a potential conflict of interest.

## Publisher’s Note

All claims expressed in this article are solely those of the authors and do not necessarily represent those of their affiliated organizations, or those of the publisher, the editors and the reviewers. Any product that may be evaluated in this article, or claim that may be made by its manufacturer, is not guaranteed or endorsed by the publisher.
