# Supplementary material for: The Potential Roles of Mucosa-Associated Invariant T Cells in the Pathogenesis of Gut Graft-Versus-Host Disease After Hematopoietic Stem Cell Transplantation
Source: Front Immunol. 2021 Sep 3;12:720354. doi: 10.3389/fimmu.2021.720354 (PMC8448388; doi:10.3389/fimmu.2021.720354)
Supplement: Supplementary file 1 [file DataSheet_1.docx]

**Supplementary** **materials**

1. **Supplementary methods**

**1.1 Patients**

The enrolled cases included the patients who received allo-HSCT for the first time in our institute and healthy donors, and the second transplantation cases were excluded. Healthy donors were required to complete pre-transplant examinations and meet the requirements of being a donor for allo-HSCT. Cases selection and samples collection were approved by the Ethics Committee of Peking University People's Hospital (ethics number 2018PHB222-01), and were carried out after obtaining the consent of the patients or legal guardian.

For flow cytometry (FCM) analysis and 16S rRNA V3-V4 region for high-throughput sequencing, the enrolled 150 consecutive patients who underwent allo-HSCT in our institute from March 1, 2019 to December 1, 2019 and met the above criteria, included 116 unmanipulated haploidentical HSCT (haplo-HSCT) and 34 HLA-matched sibling donor transplantation (MSDT) (Supplementary table 1). For in vitro stimulation experiment, 25 transplant patients hospitalized in our institute from July 10, 2020 to October 1, 2020 were enrolled, including gut aGVHD onset (8 cases), fever or infections at the same period (10 cases) and no events at the same period (7 cases) (Supplementary table 2). For in vitro co-culture experiment of MAITs and CD4+ T cells, 3 healthy adult donors were included in this study. For immunofluorescence detection, intestinal tissues were obtained from 2 healthy donors and 5 gut-aGVHD patients.

The study collected the bone marrow grafts (G-BM) and peripheral blood grafts (G-PB) of all the 150 mobilized donors. Peripheral blood samples were obtained at the time point of neutrophil engraftment, post-transplant 30 (+30), +60, +90, and +180, +270 days, and onset of gut aGVHD and after complete remission (CR). Stool samples of transplant patients were collected pre-transplant pretreatment, +14 days, onset of gut aGVHD, and after CR. In addition, the above additional enrolled 25 patients were collected PB and stool samples at the time of the event occurrence after transplantation.

**1.2 Flow cytometry**

FCM was used to detect the frequency and number of MAITs in grafts and PB, and the expression of functional factors of MAITs. In the study, MAITs were defined the group of cells that gated on CD161+Vα7.2+ in CD3+T cells (CD3+CD161+ Vα7.2+), and its frequency was defined as the ratio of CD161+Vα7.2+ to CD3+T cells (CD161+Vα7.2+/CD3+). Conventional T (Tc) and Treg cells were defined as a group of cells that expressed CD3+CD4+CD25- and CD3+CD4+CD25+CD127-, respectively.

Data from FCM were collected on the flow cytometers FACSCanto II (BD Biosciences, USA), and analyzed by FlowJo (TreeStar, USA). FCM antibodies included anti-CD3, anti-CD4, anti-CD8, anti-CD25, anti-CD69, anti-CD127, anti-CD161, anti-Vα7.2, anti-HLA-DR, anti-CCR6, anti-CXCR3, anti-CXCR4, anti-PLZF, anti-T-bet, anti-RORγt, anti-IL-17, anti-IL-22, anti-TNF-α, anti-IFN-γ, anti-GrB. The above antibodies detailed information is listed in Supplementary table 3.

**1.3 Immunofluorescence**

The staining was performed according to standard protocol (1). Intestinal tissue samples were prepared for immunofluorescence in paraffin embedding way. Briefly, paraffin-embedded samples were cut into 3-4μm sections and placed on polylysine-coated slides. Paraffin sections were baked overnight at 58°C, dewaxed in xylene, rehydrated through a graded series of ethanol, quenched for endogenous peroxidase activity in 0.3% hydrogen peroxide for 15 mins. Antigen retrieval was performed by high-pressure cooking in citrate buffer (pH=6.0) for about 20 mins, then allowed to cool to room temperature, blocking the nonspecific antibody binding sites in 5% normal goat serum for 2 hrs. Sections were stained with primary antibody CD161 (1:400,Abcam, ab197979, UK) together with CD8 (1:400, Cell Signaling Technology, 70306S, USA) at 37°C for 1.5 hrs. Biotinylated secondary antibody was performed using the EnVision+System-HRP (AEC) (K4005, Dako, Glostrup, Denmark). Subsequently, sections were counterstained with hematoxylin (Sigma-Aldrich, St Louis, MO, USA). TMA slides were scanned by an automated scanning microscope and counted by Image-Pro Plus software (IPP; produced by Media Cybernetics Corporation, USA).

**1.4 Gut microbiota Profiling**

**Sequencing**

1. Extraction of genome DNA Total genome DNA from samples was extracted using CTAB/SDS method. DNA concentration and purity were monitored on 1% agarose gels. According to the concentration, DNA was diluted to 1 ng/µL using sterile water.
2. Amplicon Generation 16S rRNA/18S rRNA/ITS genes of distinct regions (16S V4/ 16S V3/16S V3-V4/16S V4-V5, 18S V4/18S V9, ITS1/ITS2, Arc V4, et. al) were amplified used specific primer (e.g. 16S V4: 515F-806R, 18S V4: 528F-706R, 18S V9: 1380F-1510R, et. al) with the barcode. All PCR reactions were carried out in 30 µL reactions with 15 µL of Phusion® High-Fidelity PCR Master Mix (New England Biolabs); 0.2 µM of forward and reverse primers, and about 10 ng template DNA. Thermal cycling consisted of initial denaturation at 98℃for 1 min, followed by 30 cycles of denaturation at 98℃for 10 s, annealing at 50℃for 30 s, and elongation at 72℃ for 30 s. Finally, 72℃for 5 min.
3. PCR Products Mixing and Purification Mix same volume of 1×loading buffer (contained SYB green) with PCR products and operate electrophoresis on 2% agarose gel for detection. PCR products was mixed in equidensity ratios. Then, mixture PCR products was purified with GeneJETTM Gel Extraction Kit (Thermo Scientific).
4. Library preparation and sequencing Sequencing libraries were generated using Ion Plus Fragment Library Kit 48 rxns (Thermo Scientific) following manufacturer's recommendations. The library quality was assessed on the Qubit@ 2.0 Fluorometer (Thermo Scientific). At last, the library was sequenced on an Ion S5TM XL platform and 400 bp/600 bp single-end reads were generated.

**1.5 Transplantation protocol- Conditioning regimen**

Haplo-HSCT and MSDT were performed according to protocols reported previously by our group (2-3). The conditioning regimen for MSDT patients is: Cytarabine (Ara-C) 2g/m^2^/d i.v. for 1 day, cyclophosphamide (CTX) 1.8g/m^2^/d for 2 days, busulfan (BU) 0.8 mg/kg i.v., q.i.d. for 3 days, and nitrosourea (Simustine, MeCCNU) 250 mg/kg for 1 day. The conditioning regimen for haplo-HSCT patients is: Ara-C 4 g/m^2^/d i.v. for 2 days, CTX 1.8 g/m^2^/d for 2 days, BU 0.8 mg/kg i.v., q.i.d. for 3 days, and MeCCNU 250 mg/kg for 1 day, and anti-human thymus globulin (ATG, SangStat SAS, France) 2.5 mg/kg/d i.v. for 4 days. The conditioning regimen for patients with severe central nervous system invasion: TBI 770cGy, CTX 1.8 g/m^2^/d for 2 days, MeCCNU 250 mg/kg for 1 day, ATG 2.5 mg/kg/d i.v. for 4 days. The conditioning regimen for older patients with HCT-CI index greater than 3: Ara-C 2 g/m^2^/d i.v. for 2 days, CTX 1 g/m^2^/d for 2 days, BU 0.8 mg/kg i.v., q.i.d. for 3 days, Fludarabine 30mg/m^2^/d for 1 day, MeCCNU 250 mg/kg for 1 day, ATG 2.5 mg/kg/d i.v. for 4 days.

**1.6 aGVHD prevention and treatment protocols**

The aGVHD prevention protocol is a CSA/MTX/MMF combined protocol. cyclosporine A (CSA) 1.25-1.5mg/mg, q.12.h., monitor and adjust the dose according to the required concentration from 9^th^ (-9) day before transplantation. After the symptoms of the digestive tract disappear, it is changed to oral administration, and the blood concentration is maintained at 150-250ng/ml. Methotrexate (MTX) +1 day (15mg/m^2^), +3, +6, +11 day (10mg/m^2^), use calcium folinate 24 hours after MTX application. All siblings HSCT only use the first three MTX. Mycophenolate mofetil (MMF) 0.5 Bid, MSDT is discontinued after cell engraftment, non-blood HSCT is discontinued until 1 month after transplantation, and haplo-HSCT is discontinued until 2 months after transplantation.

aGVHD patients are treated in accordance with international guidelines and the specific conditions of the patients (4-5). Hormone refractory aGVHD can be diagnosed if one of the following is met, and second-line treatment can be started. After standard-dose methylprednisolone treatment: the disease progressed for 3 days; no improvement in 7 days; no cure in 14 days.

**Reference**

1. Feng Y, Li Y, Cai S, Peng J. Immunological nomograms predicting prognosis and guiding adjuvant chemotherapy in stage II colorectal cancer. Cancer Manag Res. 2019;11:7279-7294. doi: 10.2147/CMAR.S212094.
2. Huang XJ, Liu DH, Liu KY, Xu LP, Chen H, Han W, et al. Haploidentical hematopoietic stem cell transplantation without in vitro T-cell depletion for the treatment of hematological malignancies. Bone Marrow Transplant. 2006; 38(4):291–297. doi: 10.1038/sj.bmt.1705445.
3. Huang X, Liu D, Liu K, Xu L, Chen H, Han W, et al. Haploidentical hematopoietic stem cell transplantation without in vitro T cell depletion for treatment of hematologic malignancies in children. Biol Blood Marrow Transplant. 2009;15(1 Suppl):91-4. doi: 10.1016/j.bbmt.2008.10.019.
4. Martin PJ, Rizzo JD, Wingard JR, Ballen K, Curtin PT, Cutler C, et al. First- and second-line systemic treatment of acute graft-versus-host disease: recommendations of the American Society of Blood and Marrow Transplantation. Biol Blood Marrow Transplant. 2012;18(8):1150-63.   doi: 10.1016/j.bbmt.2012.04.005.
5. Dignan FL, Amrolia P, Clark A, Cornish J, Jackson G, Mahendra P, et al. Diagnosis and management of chronic graft-versus-host disease. Br J Haematol. 2012;158(1):46-61. doi: 10.1111/j.1365-2141.2012.09128.x.
6. **Supplementary Tables**

**Supplementary Table 1. Patient characteristics**

| Characteristics |  | No. of patients |
| --- | --- | --- |
| **Number** |  | 150 |
| **Median age, years** |  | 35 (14-63) |
| **Sex, male (n, %)** |  | 89 (59.3%) |
| **Diagnosis (n, %)** |  |  |
| Acute myeloid leukemia |  | 66 (44%) |
| Acute lymphoblastic leukemia  Myelodysplastic syndrome  Mixed lineage acute leukemia  Chronic myeloid leukemia  Non-Hodgkinlymphoma  Others  **ABO-matched grafts (n, %)**  Matched  Major mismatch  Minor mismatch  Bi-directional mismatch  **Donor-recipient sex-matched (n, %)**  Male-male  Male-female  Female-male  Female-female  **Pre-transplant disease status (n, %)**  CR1  CR2  CR3  NR  **Conditioning regimen (n, %)**  Bu/Cy+ATG+Ara-C+MeCCNU  Bu/Cy+Ara-C+MeCCNU  Bu/Cy+Flu+ATG+Ara-C+MeCCNU  TBI/Cy+ATG  **Transplant types**  Haploidentical  Sibling-identical  **Haploidentical patient/donor HLA compatibility**  HLA3/6  HLA4/6  HLA5/6  HLA5/10  **Stem cell source (n, %)**  G-PB  G-PB+G-BM  **Neutrophil cells engraftment (Median, days)**  **Acute GVHD grade**  I  II to IV  Gut  **Median days of post-transplant gut aGVHD onset(range)**  **Median days of post-transplant gut aGVHD CR (range)**  **Prognosis**  Relapse  Death |  | 57 (38%)  16 (10.7%)  2 (1.3%)  1 (0.6%)  3 (2.0%)  5 (3.4%)  90 (60.0%)  33 (22.0%)  21 (14.0%)  6 (4.0%)  58 (38.7%)  39 (26.0%)  31 (20.7%)  22 (14.6%)  128 (85.3%)  9 (6.0%)  1 (0.7%)  12 (8.0%)  101 (67.4%)  33 (22%)  15 (10%)  1 (0.6%)  116 (77.3%)  34 (22.7%)  93 (80.2%)  16 (13.8%)  4 (3.4%)  3 (2.6%)  17 (11.3%)  133 (88.7%)  14 (9-27)  25 (16.7%)  34 (22.7%)  16 (10.7%)  43 (27-98)  65 (40-120)  11 (7.3%)  18 (12.0%) |

**Supplementary Table 2. Clinical characteristics of MAIT in vitro activation experiment cohort**

|  | **Post-transplant patients** | | | ***P* value** |
| --- | --- | --- | --- | --- |
| **Characteristic** | **Gut aGVHD** | **Infection or fever** | **No-event** |  |
| **Number** | 8 | 10 | 7 |  |
| **Gender (male /female)** | 5/3 | 6/4 | 5/2 | 0.885 |
| **Median age, years** | 34(20-55) | 26(18-46) | 38(18-56) | 0.216 |
| **Transplant types (n, %)** |  |  |  | 0.262 |
| Haploidentical  Sibling-identical | 6(75.0%)  2(25.0%) | 10(100%)  0 | 6(85.7%)  1(14.3%) |  |
| **ABO-matched grafts (n, %)** |  |  |  | 0.774 |
| Matched | 6(75.0%) | 6(60.0%) | 5(71.4%) |  |
| Mismatched | 2(25.0%) | 4(40.0%) | 2(8.6%) |  |
| **Conditioning regimen (n, %)** |  |  |  | 0.380 |
| Bu/Cy+ATG  Bu/Cy | 6(75.0%)  2(25.0%) | 8(80.0%)  2(20.0%) | 7(100%)  0 |  |
| **Median days of the event (range)** | 43(13-61) | 36 (14-63) | 37(18-58) | 0.793 |

**Supplementary Table 3. FCM antibody list.**

| Antibody | Channel | Company | Clone |
| --- | --- | --- | --- |
| CD3 | PE-Cy7 | Biolegend | UCHT1 |
| CD3 | Percp | Biolegend | SK7 |
| CD4 | Percp | Biolegend | OKT4 |
| CD4 | APC-Cy7 | BD | RPA-T4 |
| CD8 | APC-R700 | BD | RPA-T8 |
| CD25 | BV605 | BD | 2A3 |
| CD69 | BV510 | Biolegend | FN50 |
| CD127 | BV510 | BD | 7R-M21 |
| CD161 | APC | Biolegend | HP-3G10 |
| CD161 | PE-Cy7 | Biolegend | HP-3G10 |
| TCR Vα7.2 | PE | Biolegend | 3C10 |
| TCR Vα7.2 | APC | Biolegend | 3C10 |
| HLA-DR | BV421 | Biolegend | L243 |
| CCR6 | FITC | Biolegend | G034E3 |
| CXCR3 | BV605 | Biolegend | G025H7 |
| CXCR4 | APC-Cy7 | Biolegend | 12G5 |
| PLZF | APC | R&D | / |
| T-bet | AF488 | BD | O4-46 |
| RORγt | BV421 | BD | Q21-559 |
| IL-17 | PE | BD | N49-653 |
| IL-22 | FITC | Biolegend | 2G12A41 |
| TNF-α | BV605 | Biolegend | MA611 |
| IFN-γ | BV510 | Biolegend | 4S.B3 |
| Granzyme B | BV421 | Biolegend | QA18A28 |

**Supplementary Table 4. Characteristics of donors and graft composition analysis from corresponding recipients with gut aGVHD, skin aGVHD and no aGVHD.**

| **Donors**  **Characteristics** | **Gut aGVHD**  **N=16** | **Skin aGVHD**  **N=45** | **No aGHVD**  **N=89** | ***P^*^*** | ***P^**^*** | ***P^***^*** |
| --- | --- | --- | --- | --- | --- | --- |
| **Donors**  Gender (male/female)  Weight (kg)^a^  **G-BM harvesting** | 13/3  60.0 (35.1-83.0) | 32/13  60.1 (41.0-97.5) | 52/37  60.1 (40.0-89.3) | .157  .182 | .084  .665 | .832  .132 |
| Tc (×10^6^ /kg)^a^  Treg (×10^6^ /kg)^a^  MAIT (×10^6^ /kg)^a^  CD34 (10^6^/Kg) | 6.53 (1.79-23.04)  0.12 (0.03-0.38)  0.54 (0.05-3.46)  0.54 (0.23-1.33) | 5.53 (1.14-13.02)  0.08 (0.01-0.28)  0.45 (0.07-2.84)  0.41 (0.11-1.51) | 5.61 (0.67-26.04)  0.09 (0.01-0.59)  0.53 (0.07-3.39)  0.54 (0.05-1.55) | .285  .772  .817  .587 | .192  .184  .103  **.043** | .070  .264  .199  .077 |
| **G-PB harvesting** |  |  |  |  |  |  |
| Tc (×10^6^ /kg)^a^  Treg (×10^6^ /kg)^a^  MAIT (×10^6^ /kg)^a^  CD34 (10^6^/kg) ^a^  **Mixture grafts**  Tc (×10^6^ /kg)^a^  Treg (×10^6^ /kg)^a^  MAIT (×10^6^ /kg)^a^  CD34 (10^6^/kg) ^a^  CD4/CD8  MNC (10^8^/kg) ^a^ | 116.43(30.89-232.61)  2.29 (0.29-8.14)  3.81 (0.93-14.22)  2.03 (0.17-6.12)  128.78(53.93-232.61)  2.31 (0.36-8.17)  4.22 (0.93-15.50)  2.45 (1.00-6.12)  1.38 (0.71-2.88)  8.68 (7.32-12.01) | 89.75(0.14-272.49)  1.93 (0.06-7.51)  4.43 (0.01-28.05)  2.10 (0.34-7.97)  97.11(8.72-285.51)  1.96 (0.20-7.75)  4.57 (0.11-30.90)  2.56 (0.56-8.30)  1.24 (0.41-5.37)  8.82 (5.95-20.23) | 104.69(0.41-389.67)  2.04 (0.03-12.41)  4.99 (0.05-52.57)  2.12 (0.38-7.00)  111.05(4.5-389.67)  2.09 (0.06-12.41)  5.62 (0.31-54.55)  2.67 (0.85-19.00)  1.18 (0.47-4.92)  8.69 (5.21-14.33) | .523  .383  **.003**  .750  .509  .362  **.005**  .587  .386  .912 | .244  .959  .150  .256  .230  .984  .163  .145  .217  .273 | .175  .405  .440  .657  .161  .395  .663  .653  .979  .979 |

^a^Data are reported as median (range).

*P^*^*-value between the donor graft of corresponding recipients with gut aGVHD and no aGVHD.

P**-value between the donor graft of corresponding recipients with skin aGVHD and no aGVHD.

*P^***^*-value between the donor graft of corresponding recipients with skin aGVHD and gut aGVHD.

*P*＜0.05 was marked in bold.

**Supplementary Table 5. Characteristics of patients with gut aGVHD and Chemotherapy- or infection-induced diarrhea after transplantation.**

| **Characteristics** | **Gut aGVHD** | **Chemotherapy- or infection-induced diarrhea** | ***P* value** |
| --- | --- | --- | --- |
| **Number** | 16 | 11 |  |
| **Median age, years** | 27(16-59) | 37(16-56) | 0.226 |
| **Sex, male (n, %)** | 12(75.0%) | 8(72.8%) | 0.895 |
| **ABO-matched grafts (n, %)** |  |  | 0.096 |
| Match | 5(31.3%) | 7(63.6%) |  |
| Dismatch | 11(68.7%) | 4(36.4%) |  |
| **Conditioning regimen (n, %)**  Bu/Cy+ATG  Bu/Cy  Bu/Cy+Flu+ATG | 12(75.0%)  3(18.7%)  1(6.3%) | 8(72.8%)  1(9.1%)  2(18.1%) | 0.535 |
| **Transplant types**  Haploidentical  Sibling-identical | 13(81.3%)  3(18.7%) | 10(90.9%)  1(9.1%) | 0.488 |

**Supplementary Table 6. Univariate and multivariate analyses of risk factors for the occurrence of aGVHD**

|  | **Univariate analyses** | | | **Multivariate analyses** | | |
| --- | --- | --- | --- | --- | --- | --- |
| **Characteristics** | HR | 95%CI | *P* value | HR | 95%CI | *P* value |
| Donor gender | 0.562 | 0.312-1.010 | **0.054** | 0.736 | 0.309-1.758 | 0.491 |
| Donor ages | 1.020 | 1.002-1.039 | **0.031** | 1.020 | 0.995-1.045 | 0.117 |
| Recipient ages | 0.982 | 0.963-1.002 | **0.074** | 0.994 | 0.967-1.021 | 0.641 |
| Recipient gender | 0.837 | 0.494-1.418 | 0.508 |  |  |  |
| ABO-matched grafts | 1.202 | 0.719-2.009 | 0.484 |  |  |  |
| disease status （pre-transplant CR vs. NR） | 1.073 | 0.461-2.496 | 0.870 |  |  |  |
| Transplant types | 0.369 | 0.167-0.812 | **0.013** | 0.500 | 0.029-8.606 | 0.633 |
| Donor-recipient gender match | 0.937 | 0.677-1.296 | 0.693 |  |  |  |
| Conditioning regimen | 0.605 | 0.384-0.952 | **0.030** | 1 |  | 0.557 |
| G-BM CD34+counts | 0.527 | 0.219-1.271 | 0.154 |  |  |  |
| G-BM Treg counts | 0.212 | 0.008-5.711 | 0.356 |  |  |  |
| G-BM Tc counts | 0.981 | 0.911-1.056 | 0.610 |  |  |  |
| G-BM MAIT counts | 0.761 | 0.468-1.293 | 0.273 |  |  |  |
| G-PB CD34+counts | 0.915 | 0.770-1.087 | 0.311 |  |  |  |
| G-PB Treg counts | 1.018 | 0.903-1.147 | 0.774 |  |  |  |
| G-PB Tc counts | 0.998 | 0.993-1.003 | 0.382 |  |  |  |
| G-PB MAIT counts | 0.958 | 0.915-1.002 | **0.059** | 0.959 | 0.884-1.040 | 0.310 |
| Graft CD34+ counts | 0.901 | 0.773-1.051 | 0.183 |  |  |  |
| Graft Treg counts | 1.020 | 0.906-1.148 | 0.744 |  |  |  |
| Graft Tc counts | 0.998 | 0.993-1.003 | 0.372 |  |  |  |
| Graft CD4/CD8 | 1.282 | 0.940-1.750 | 0.117 |  |  |  |
| Graft MNC | 1.076 | 0.943-1.228 | 0.275 |  |  |  |
| Graft MAIT counts（≥5.3×10^6^/Kg（median value）vs. <5.3×10^6^/Kg | 0.616 | 0.348-1.092 | **0.097** | 0.381 | 0.158-0.915 | **0.031** |
| Day +30 MAIT counts（≥1.1/μL（median value）vs. <1.1/μL | 0.679 | 0.376-1.227 | 0.199 |  |  |  |
| Day +60 MAIT conuts（≥2.9/μL（median value）vs. <2.9/μL | 0.538 | 0.295-0.984 | **0.097** | 0.734 | 0.315-1.714 | 0.475 |

The factors with *P*<0.1 in univariate analysis were included in multivariate analysis

Graft bone marrow (G-BM); Graft peripheral blood (G-PB)

**3 Supplementary Figures**

**A**

**B**

**C**


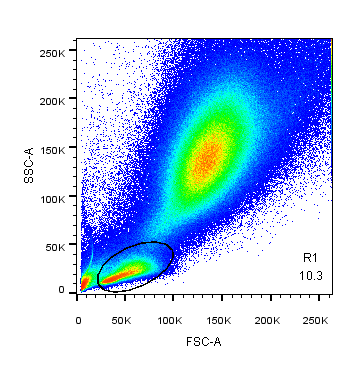

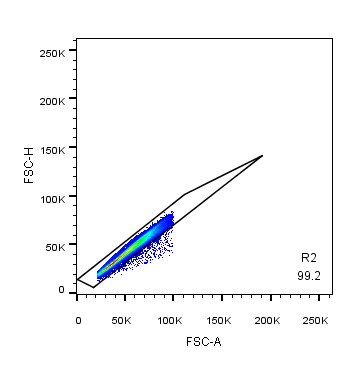

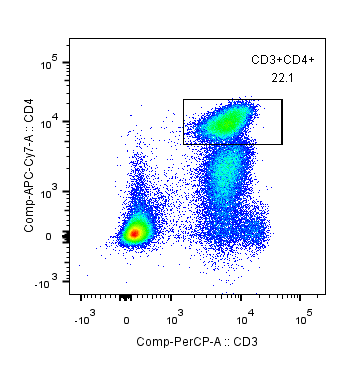

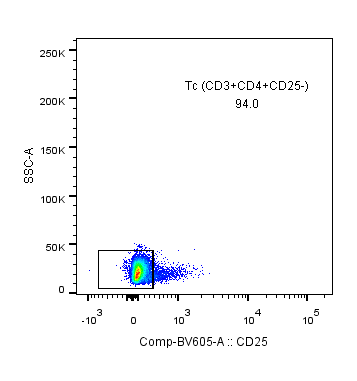

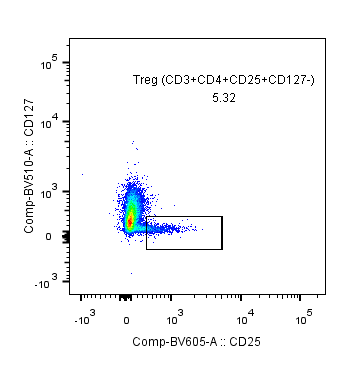


**D**

**E**


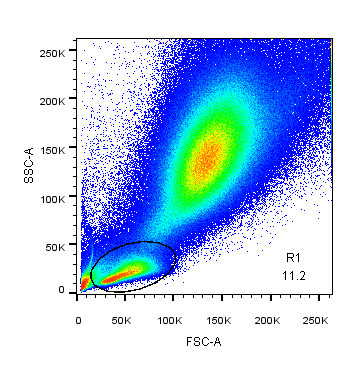

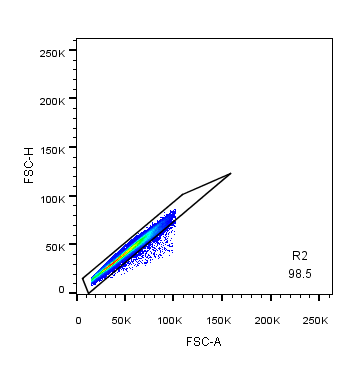

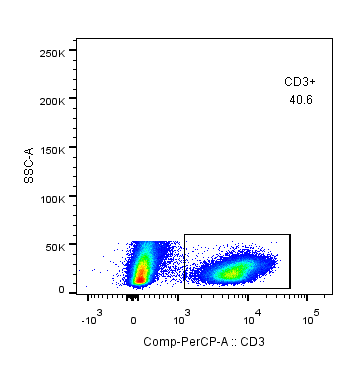

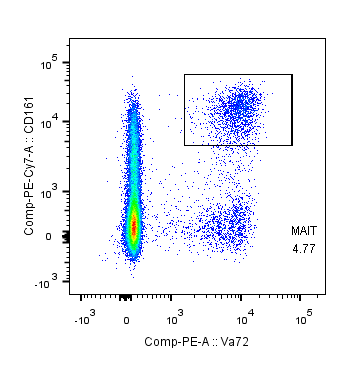

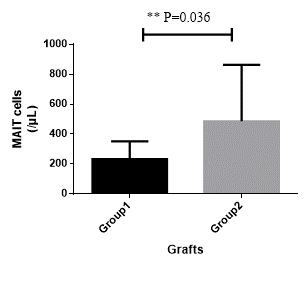

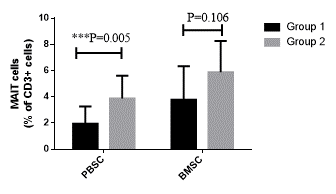


**CD3**

**Vα7.2**

**CD161**

**Supplementary Figure 1** Comparison of the frequency and number of MAIT cells in the grafts from the rhG-CSF mobilized donors and healthy person. **(A)** MAIT cell (CD3+CD161^hi^Vα7.2) staining and analyzing strategy by FCM in blood samples. **(B)** Conventional T cells (CD3+CD4+CD25-) and Treg cells (CD3+CD4+CD25+CD127-) staining and analyzing strategy by FCM in blood samples. **(C)** The frequency of conventional T cells (% of CD3+CD4+ cells) in graft-BM and graft-PB**. (D)** The frequency of Treg cells (of CD3+CD4+ cells) in graft-BM and graft-PB**. (E)** The frequency of MAIT cells in graft-BM and graft-PB (*P*=0.005), and the number of MAIT cells in total grafts (*P*=0.036) from gut aGVHD group (group 1, n=16) and chemotherapy- or infection-induced diarrhea group (group 2, n=11). Levels of significances are given as *P*-values with ***≤0.01 and **≤0.5.


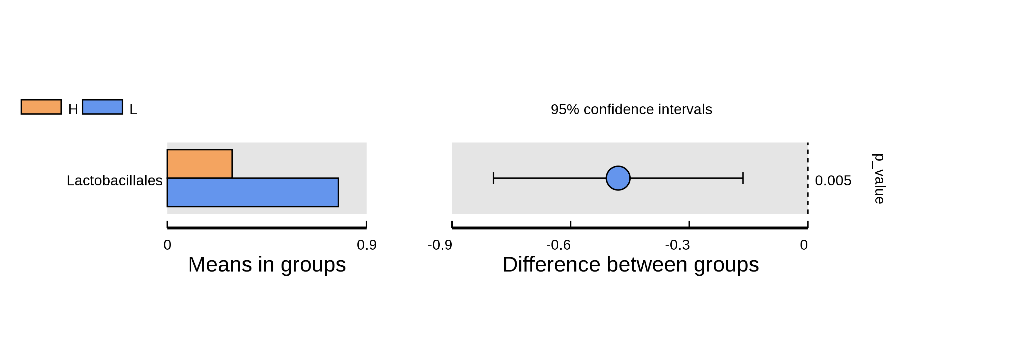


**Order**


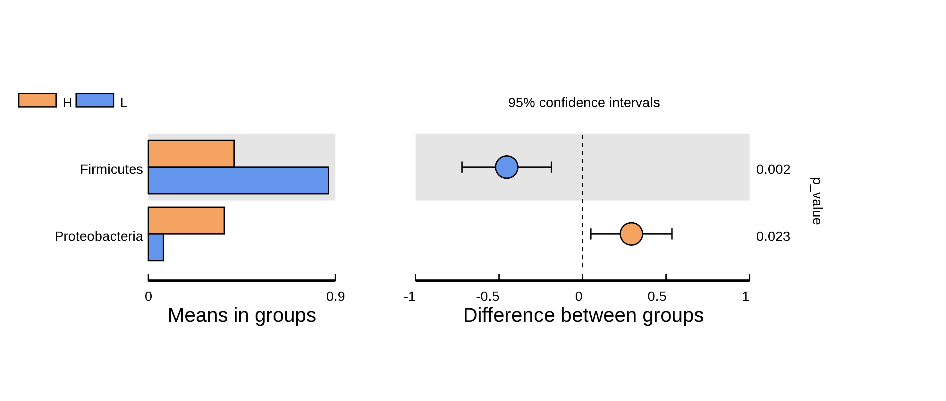


**Phylum**

Groupgr

**Supplementary Figure 2** T-test species difference analysis diagram at the order and phylum levels between high number of MAIT cells in infused grafts (> 5.3×10^6^/kg, H group, n=12) and low number of MAIT cells (<5.3×10^6^/kg, L group, n=10) in infused grafts.

**A**

**B**


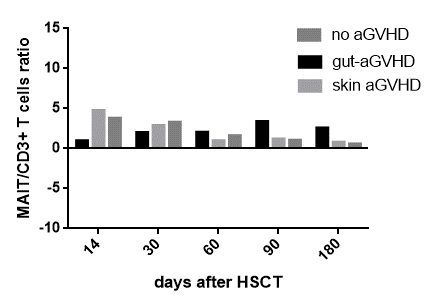

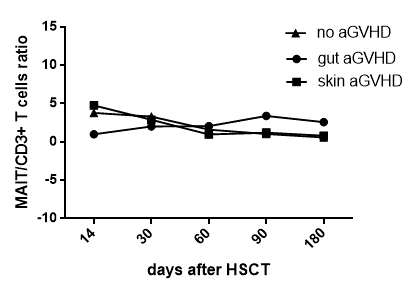


**Supplementary Figure 3** Reconstruction of MAIT cells and their different subgroups after transplantation. **(A)** Post-transplant the frequency of MAIT cells reconstruction under haplo-HSCT and sibling-identical HSCT. **(B)** Post-transplant the frequency of MAIT cells (% CD3+ T cells) reconstruction in gut aGVHD patients, skin aGVHD patients and no gut aGVHD patients.


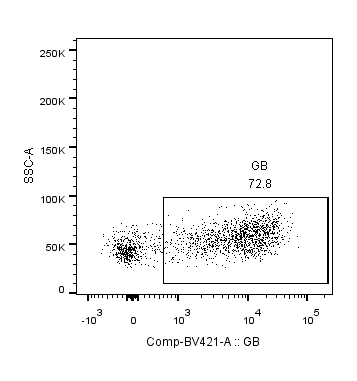

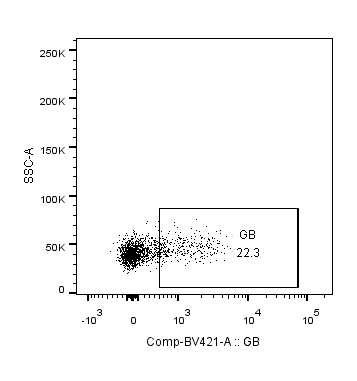


**CD3+CD28**


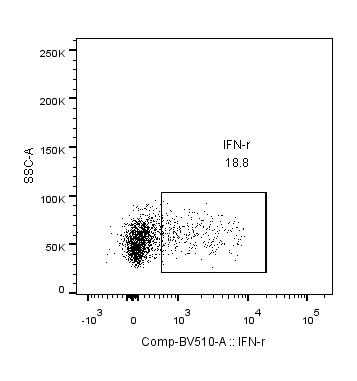

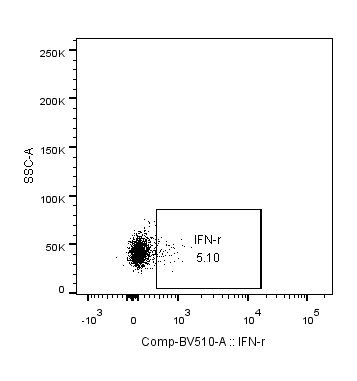


**IL-12+1L-18**

**CD3+CD28**


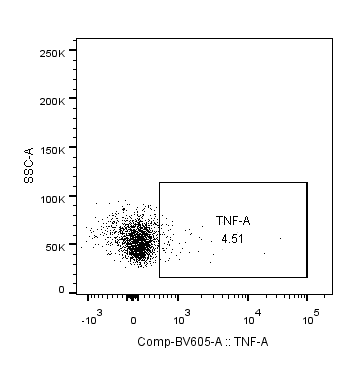

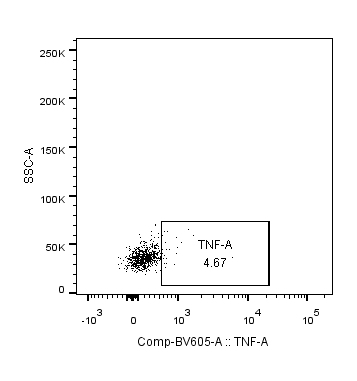

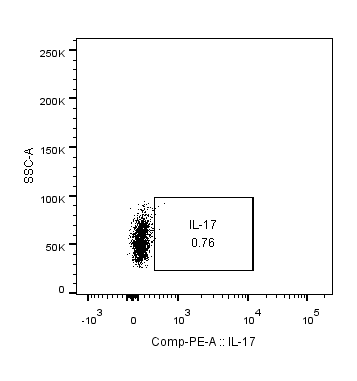

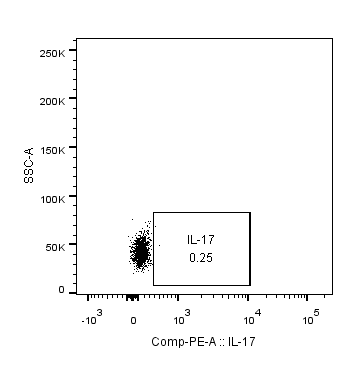

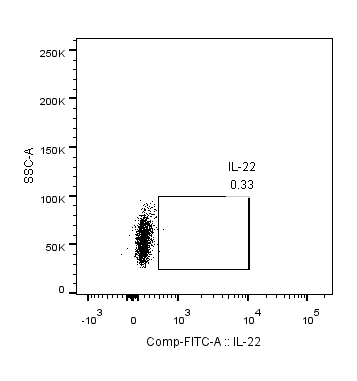

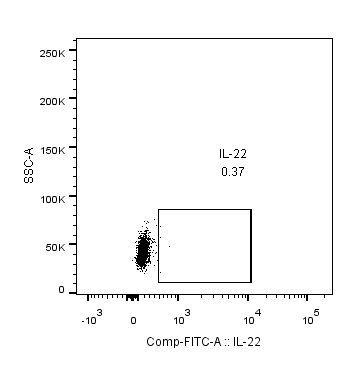


**IL-12+1L-18**

**IL-12+1L-18**

**IL-12+1L-18**

**CD3+CD28**

**CD3+CD28**

**CD3+CD28**

**Granzyme**

**Granzyme**

**IFN-γ**

**IFN-γ**

**TNF-α**

**TNF-α**

**IL-17**

**IL-17**

**IL-22**

**IL-22**

**A**

**B**

**IL-12+1L-18**

**C**


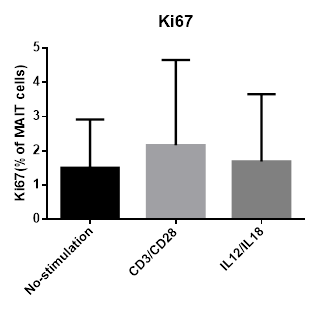


**D**

**Supplementary Figure 4 (A) and (B)** Flow cytometry of cytokine expression of MAIT in peripheral blood under IL-12/IL-18 stimulation and CD3/CD28 stimulation *in vitro* from healthy donors. **(C)** Expression of the MAIT proliferation marker Ki67 under no-stimulation, IL-12/IL-18 stimulation and CD3/CD28 stimulation *in vitro* from healthy donors **(D)** The comparison of cytokines expression under IL-12/IL-18 stimulation and CD3/CD28 stimulation in each MAIT subset.

**Gated on CD3+ cells**

**A**

**B**

**C**


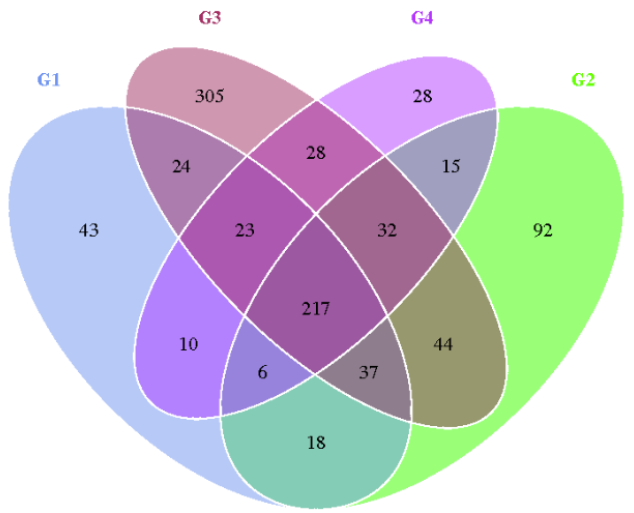

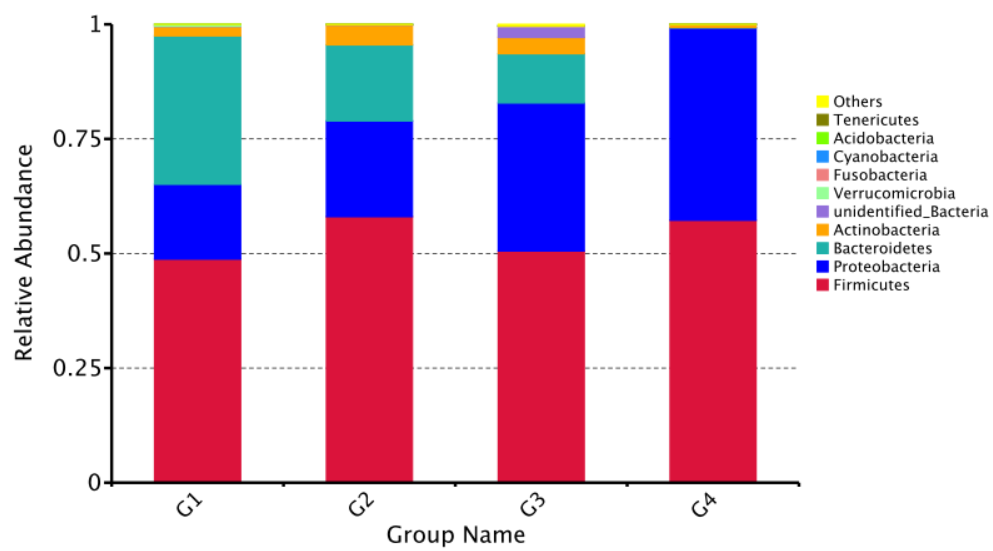


**D**

**G1: Pre-transplant**

**G2: Engraftment**

**G3: Gut aGVHD**

**G4: CR**


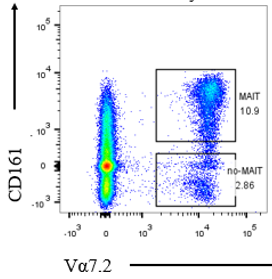


**Supplementary Figure 5** MAIT cell functional changes in patients with gut aGVHD before and after the onset of gut aGVHD. **(A)** The changes of no-MAIT cells (CD3+CD161-Vα7.2) number in 16 patients with gut aGVHD at three post-transplant time points, namely at the time of neutrophil engraftment, at the onset of gut aGVHD, and at the CR of gut aGVHD. **(B)** The changes of transcription factors PLZF, T-bet and Rorγt in patients with gut aGVHD at different points. **(C)** The expression of markers and factors at the onset of gut aGVHD in the MAIT subgroups divided by CD4 and CD8. **(D)** Venn diagram and the column chart of relative abundance of species at phylum level.


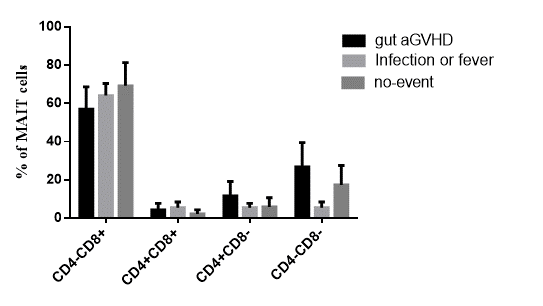


**B**

**C**

**A**

**Supplementary Figure 6** **(A)** The comparison of the frequency of MAIT cells among gut aGVHD group, infection or fever group and no-event group (Frequency: Gut aGVHD vs. No-event, *P*<0.001). **(B)** The comparison of the transcription factors among the three groups (Rorγt: Gut aGVHD vs. No-event, *P*=0.037; T-bet: Gut aGVHD vs. No-event, *P*=0.018; Gut aGVHD vs. Infection or fever, *P*=0.023). **(C)** The comparison of the CD4 and CD8 phenotypic groups among the three groups. Levels of significances are given as *P*-values with ***≤0.01 and **≤0.5.


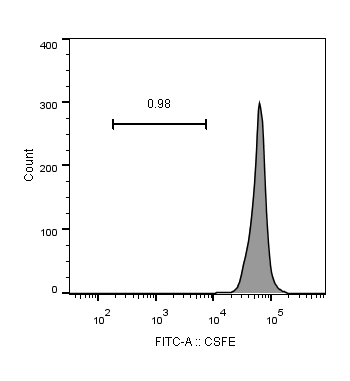

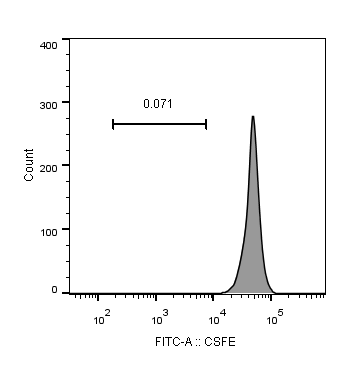

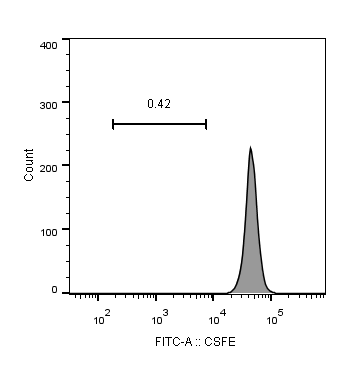

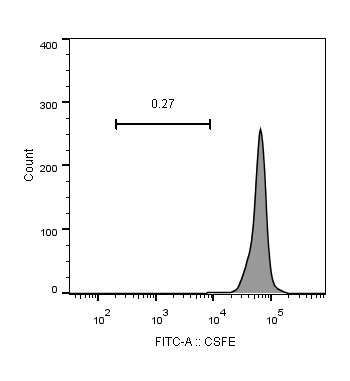

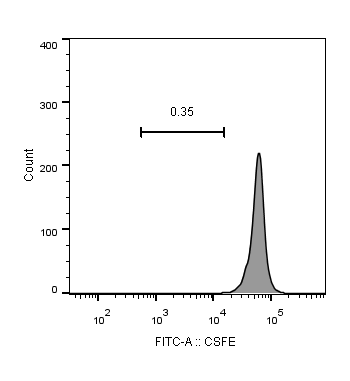

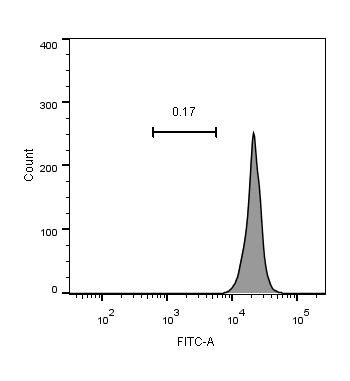

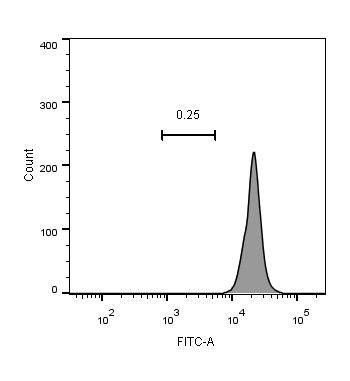


**MAIT : Tc**

**T : Tc**

**2:1**

**1:1**

**1:1**

**2:1**

**1:2**

**1:2**

**1:4**

B

**CFSE**


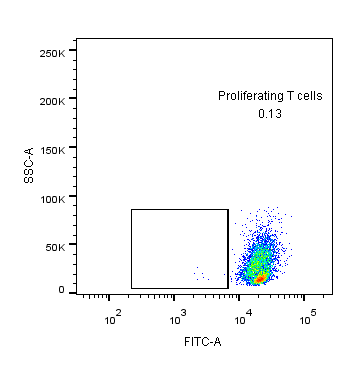

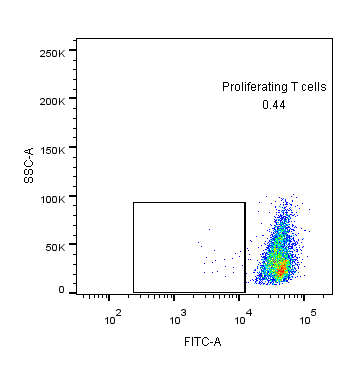


C

**MAIT : Tc**

**CFSE**

**T : Tc**


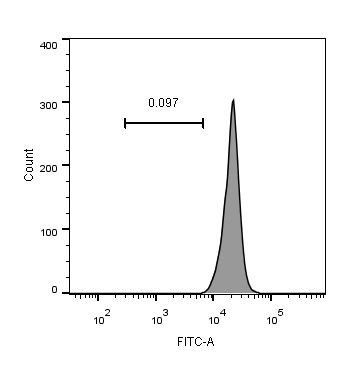


**1:4**

**A**


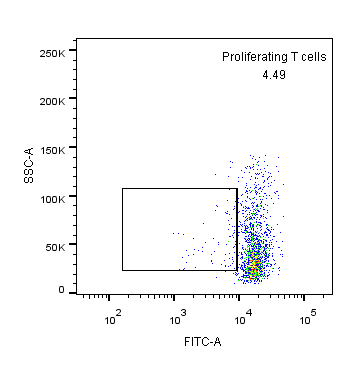

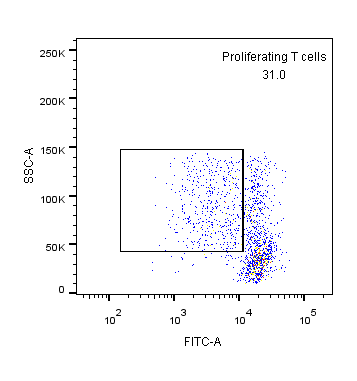

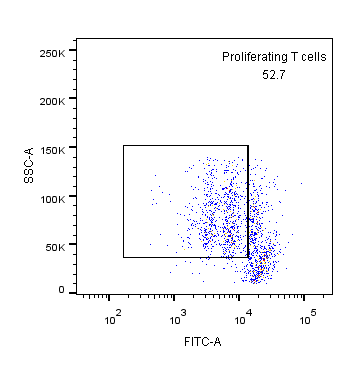

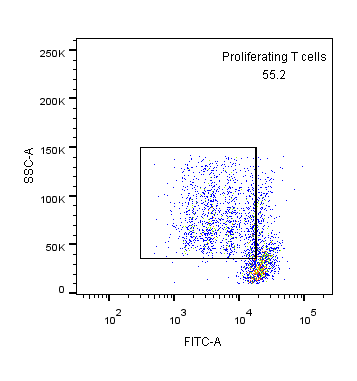

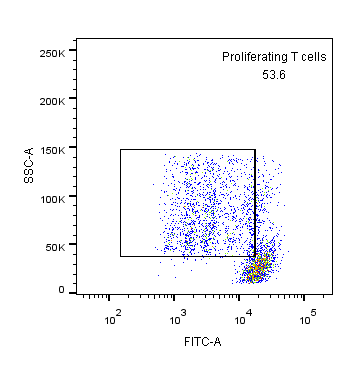

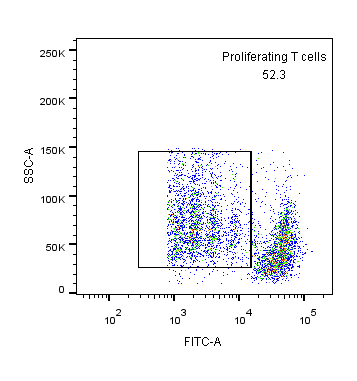

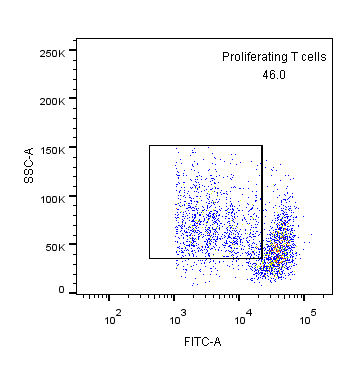

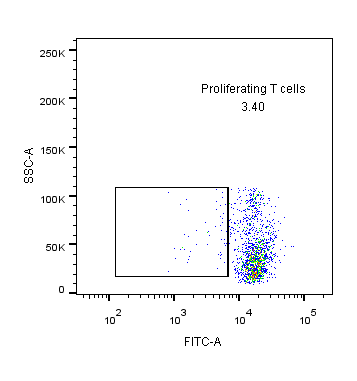


**2:1**

**1:1**

**1:1**

**2:1**

**1:2**

**1:2**

**1:4**

**1:4**

**MAIT : Tc**

**Tc : Tc**

**CFSE**

**Supplementary Figure 7** The vitro inhibition experiment of MAIT on CD4+T cells. **(A)** MAIT and CD4 + T cells were isolated from the peripheral blood of 4 healthy donors. CFSE-labeled CD4 + T cells with CD3/CD28 beads and MAIT or CD4 + T cells were mixed and cultured at a ratio of 2:1, 1:1, 1:2, and 1:4 for 4 days. Representative flow cytometric analysis of CFSE dilutions in CD4 + T cells. **(B)** and **(C)** MAIT and CD4 + T cells were isolated from the peripheral blood of 4 healthy donors. CFSE-labeled CD4 + T cells without CD3/CD28 beads and MAIT cells or CD4 + T cells were mixed and cultured at a ratio of 2:1, 1:1, 1:2, and 1:4 for 4 days. Representative flow cytometric analysis of CFSE dilutions in CD4 + T cells.

**Supplementary Figure 8**

B

**A**

**Supplementary Figure 8 (A)** Comparison of CD8 staining intensity, CD8 positive cell ratio, number and density between healthy person intestinal tissue (n=2) and intestinal lesion tissue of gut aGVHD patients (n=5). **(B)** Comparison of CD8+CD161- cell ratio, number and density between healthy person intestinal tissue (n=2) and intestinal lesion tissue of gut aGVHD patients (n=5).
